# Alcohol exposure suppresses ribosome biogenesis and causes nucleolar stress in cranial neural crest cells

**DOI:** 10.1371/journal.pone.0304557

**Published:** 2024-06-28

**Authors:** George R. Flentke, Thomas E. Wilkie, Josh Baulch, Yanping Huang, Susan M. Smith

**Affiliations:** 1 UNC Nutrition Research Institute, University of North Carolina at Chapel Hill, Kannapolis, NC, United States of America; 2 Department of Nutrition, University of North Carolina at Chapel Hill, Kannapolis, NC, United States of America; University of Louisville, UNITED STATES

## Abstract

Prenatal alcohol exposure (PAE) causes cognitive impairment and a distinctive craniofacial dysmorphology, due in part to apoptotic losses of the pluripotent cranial neural crest cells (CNCs) that form facial bones and cartilage. We previously reported that PAE rapidly represses expression of >70 ribosomal proteins (padj = 10-E47). Ribosome dysbiogenesis causes nucleolar stress and activates p53-MDM2-mediated apoptosis. Using primary avian CNCs and the murine CNC line O9-1, we tested whether nucleolar stress and p53-MDM2 signaling mediates this apoptosis. We further tested whether haploinsufficiency in genes that govern ribosome biogenesis, using a blocking morpholino approach, synergizes with alcohol to worsen craniofacial outcomes in a zebrafish model. In both avian and murine CNCs, pharmacologically relevant alcohol exposure (20mM, 2hr) causes the dissolution of nucleolar structures and the loss of rRNA synthesis; this nucleolar stress persisted for 18-24hr. This was followed by reduced proliferation, stabilization of nuclear p53, and apoptosis that was prevented by overexpression of MDM2 or dominant-negative p53. In zebrafish embryos, low-dose alcohol or morpholinos directed against ribosomal proteins *Rpl5a*, *Rpl11*, and *Rps3a*, the *Tcof* homolog *Nolc1*, or *mdm2* separately caused modest craniofacial malformations, whereas these blocking morpholinos synergized with low-dose alcohol to reduce and even eliminate facial elements. Similar results were obtained using a small molecule inhibitor of RNA Polymerase 1, CX5461, whereas p53-blocking morpholinos normalized craniofacial outcomes under high-dose alcohol. Transcriptome analysis affirmed that alcohol suppressed the expression of >150 genes essential for ribosome biogenesis. We conclude that alcohol causes the apoptosis of CNCs, at least in part, by suppressing ribosome biogenesis and invoking a nucleolar stress that initiates their p53-MDM2 mediated apoptosis. We further note that the facial deficits that typify PAE and some ribosomopathies share features including reduced philtrum, upper lip, and epicanthal distance, suggesting the facial deficits of PAE represent, in part, a ribosomopathy.

## Introduction

Ribosomopathies represent disorders–mostly genetic in origin–caused by an impairment of the ribosome biogenesis (RBG) that is necessary to replenish the pool of ribosomes following each cell division [1, 2]. Ribosomopathies are typically diagnosed by impairments in cell division that often present as anemia [3, 4]. Others can also feature additional phenotypes including growth stunting, cognitive deficits, and a distinctive craniofacial appearance, and the basis for these diverse phenotypes among ribosomopathies is not understood [5, 6].

The replenishment of ribosomes through RBG is estimated to consume up to 70–80% of the cell’s energy budget [7], and this high demand can be literally visualized within the nucleus as the nucleolar structures, which represent the sites of rRNA transcription by RNA polymerase I and III, its processing by >100 enzymes, and its assembly with the ribosome proteins (RPs) [8, 9]. The demand is so high that rRNA is encoded in multiple locations across the genome, and cells can fine-turn RBG by silencing some sites and not others [10]. Given its high energy demand, RBG is tightly linked to cellular energy status through the regulatory mediators TORC1 and AMPK [11]. Cells actively monitor RBG as an indicator of cellular stress. Such stress is manifested by a reduction in rRNA and RP transcription and is seen visually as a dissolution of the nucleolar structure where RBG occurs [12–16]. Also known as nucleolar stress, under this reduction in rRNA content select RPs including RPL5, RPL11, RPS3, and RPL37, instead interact with and divert the nuclear E3 ubiquitinase murine double minute (MDM2) [17–20]. Under normal conditions MDM2 ubiquitinates p53, targeting the latter for proteasomal destruction. Under nucleolar stress, the RP-MDM2 interaction permits the stabilization of p53 which then acts to suppress cell division. Under severe stress the active p53 will also initiate the cell’s apoptosis.

One cell lineage that has higher sensitivity to nucleolar stress and apoptosis is the cranial neural crest (CNC), a stem cell lineage that emerges early in embryogenesis to form the facial bone and connective tissue, among other structures [21]. Several ribosomopathies are characterized by facial deficits including Treacher-Collins syndrome, Diamond-Blackfan anemias, Roberts syndrome, and acrofacial dysostosis [5, 6, 22]. Cellular-level studies reveal that the loss of select RPs that interact with MDM2, or the loss of enzymes critical for rRNA synthesis and processing, initiates the p53-mediated apoptosis of CNC progenitors to produce facial deficits [23–25]. The basis for the CNC’s sensitivity is unclear but may be related to their need to delaminate and migrate into the facial anlage, a process that is tightly regulated and is coordinated with their rapid proliferation [26, 27].

We have observed that several of the facial deficits that characterize ribosomopathies, including absent philtrum, thin upper lip, epicanthal folds, and micrognathia, are also diagnostic for Fetal Alcohol Spectrum Disorders (FASD), an umbrella term that encompasses the craniofacial, growth, and neurobehavioral and cognitive deficits that can result from prenatal alcohol exposure (PAE) [28]. We and others have reported that at pharmacologically-relevant concentrations (0.05–0.3 mg%; 12–50 mM), alcohol exposure causes the apoptosis of neural crest progenitors [29–31]. This apoptosis is accompanied by the stabilization of p53, and blockade of p53 activity by molecular and genetic means prevents CNC cell death [32–34]. Transcriptome analysis provided mechanistic additional insight and 6hr following alcohol exposure (52mM) the most significantly altered pathway in these early progenitors was Ribosome Protein (KEGG #04150, padj = 10E-47), with a 20–70% suppression of 76 cellular and mitochondrial RPs [35]. This same pathway also had the greatest underrepresentation in the expression-level comparison of alcohol-vulnerable versus -resistant neural crest lineages [36].

These points of similarity led us to hypothesize that the alcohol-induced apoptosis of CNC involves nucleolar stress. Here we show that alcohol causes nucleolar stress in both primary CNCs and a CNC-derived cell line, and this is followed by the downstream activation of MDM2-dependent p53-mediated signaling. These data suggest that the alcohol-mediated apoptosis of CNCs and the subsequent facial deficits that characterize those with FASD represent, in part, a ribosomopathy.

## Materials and methods

### O9-1 cranial neural crest cell line model

O9-1 cells were obtained from Millipore (#SCC049; Burlington, MA). They were originally isolated from mass cultures of primary CNCs that express Wnt1-Cre:R26R-GFP, and were derived from C57BL/6J mouse embryos at embryonic day 8.5 [37]. These cells express key CNC markers (e.g., Twist1, Snail1, Nestin, CD44) and can differentiate into osteoblasts, chondrocytes, smooth muscle cells, and glial cells, but not neuronal cells [37]. Cells were cultured on Complete ES Cell Media (#ES-101-B, Millipore) supplemented with 100 U/ml penicillin, 100 U/ml streptomycin, and 25ng/ml basic fibroblast growth factor (bFGF; Invitrogen, Waltham, MA). Some experiments were done on a low (1% FCS) conditioned media that maintained pluripotent cell status using 92% DMEM (Gibco), 6% Complete ES Cell Media, 0.1mM nonessential amino acids, 1 mM sodium pyruvate, 55mM β-mercaptoethanol, 100 U/ml penicillin, 100 U/ml streptomycin, 2mM L-glutamine, and supplemented for O9-1 use with bFGF and 1000 U/ml leukemia inhibitory factor (LIF, Gibco; Grand Island NY). This media was used for 24 hours or less. The alcohol exposure used USP-grade 100% ethanol (Koptec, King of Prussia, PA) at concentrations between 0 to 100mM for 2 to 24 hours.

### Primary cranial neural crest model

Studies involving these early stages of chick embryos do not require review by the UNC Animal Care and Use Committee. Fertile chicken eggs (strain Hampshire Red, Department of Poultry Science, North Carolina State University, Raleigh NC) were incubated to the 4–8 somite stage. The early crania were dissected free and incubated in F12 containing 10% fetal calf serum (heat-inactivated) and 7.5% Chick Embryo Extract (prepared from 10-day-old embryos as per) [38, 39], and 1× penicillin-streptomycin, and containing alcohol concentrations from 0 to 80 mM. After 2 hr, isolated crania were gently washed several times in alcohol-free media and immediately explanted as below.

Primary CNCs were prepared from the above crania using explant cultures as described [32]. In brief, isolated crania from above were placed dorsal side down atop glass coverslips that had been pretreated with bovine plasma fibronectin (25 ug/ml, Invitrogen), then incubated (37°C under 5% CO2) in F12 medium containing 10% heat-inactivated fetal bovine serum, 1× penicillin-streptomycin, and 7.5% chick embryo extract. After 18 hr incubation, cranial tissue was gently lifted away using fine forceps and migrated cells were fixed in 4% paraformaldehyde in phosphate buffered saline (PBS) for subsequent immunostaining and cell quantitation.

### Zebrafish model

Studies using zebrafish were approved by the University of North Carolina at Chapel Hill Animal Care and Use Committee (22–079.0-A) and were performed in our AAALAC-approved facility at the UNC NRI. Wild-type zebrafish (*D*. *rerio*) of the 5D strain were provided by R. Tanguay (Oregon State University, Corvallis OR) and were housed under standard conditions (28°C, 14hr/10hr light-dark cycle). Fertile eggs were harvested within 30min of timed spawns and washed in Embryo Media (EM). At 70% epiboly, chorionated embryos were exposed for 6hr to alcohol diluted in EM [31], and then washed and returned to incubation. The tests for synergism between alcohol and RBG loss-of-function used 252mM in the EM (lower dose), which we showed previously causes only modest craniofacial deficits [31, 35]. The test of p53 morpholinos to rescue the alcohol-induced facial deficits used 500mM in the EM (high dose) [31]. However, because only 36% of the external ethanol content crosses the chorion to enter the embryo [31, 40], the embryo’s true alcohol exposure is 90mM and 172mM, respectively. At 4- or 5-days post-fertilization (dpf) the embryos were euthanized by holding on wet ice for 20min, fixed, and stained to visualize cartilage using 0.05% Alcian Blue (Sigma, A3157) [35]. Studies of the RNA Polymerase 1 inhibitor CX5461 used embryos that were first dechorionated using pronase (0.1 mg/ml for 3-15min; Sigma) as per [31], rinsed, treated with CX5461 (10 μM, Selleck Chemicals, Houston TX) for 5.5hrs, rinsed again, and finally exposed to 90mM alcohol in EM for 6hr and processed as above.

## Whole transcriptome analysis

This utilized the chick whole transcriptome dataset previously published by our lab [35] and is comprised of sequences generated from chick embryo neural folds having five to eight somites (pool from 23 embryos, somite-matched), and isolated 6 hr following their exposure to alcohol (50–60 mM in ovo, 90 minutes) or isotonic saline. The cDNA sequences were 75-bp paired-end reads (Illumina Genome Analyzer IIx, University of Wisconsin-Madison Biotechnology Center), and analyzed using the previously described filtering processes, alignment, and mapping to the *Gallus gallus* genome (Galgal4 e73; [35, 36]. P-values were adjusted for the false discovery rate using the Benjamini-Hochberg multiple testing correction. Because the *G*. *gallus* genome is not well-annotated with respect to KEGG, we visually extracted from this dataset genes within KEGG pathways related to ribosome activity including rRNA Synthesis (#03020), rRNA Processing and Assembly (#03008), Ribosomal Proteins (#03010), tRNA Synthesis (#00970), and the protein and ribosome synthesis aspect of mTOR Signaling Pathway (#04150). We separately searched the gene list for genes within the P53 signaling pathway (#04115).

### Immunostaining

All samples were fixed with 4% paraformaldehyde in PBS for 15 min at RT or 4 hours 4°C. Samples were then washed 3 times with PBS containing 0.05% Tween-20 (PBST), and dehydrated in graded ethanol solutions (30%, 50%, 2×70%, 85%) and stored at 4°C until used. For analysis, samples were rehydrated using the reverse ethanol series, equilibrated 3× in PBST, and then blocked overnight at 4°C in PBST containing 1% heat-inactivated goat serum (Gibco) and 1% BSA (Thermo-Fisher, Waltham, MA) in PBST. The primary antibodies, sources, and dilutions are presented in **S1 Table**. Isotype-specific secondary antibodies (Southern Biotech, Birmingham, AL) coupled to Alexa-488 (chicken cells) or Alexa594 (mouse cells) were used at 1:1000. Cells were counter-stained with DAPI to visualize nuclei (0.5μg/ml, Southern Biotech). Digital images were created using fixed exposure, and the fluorescent signal per cell was quantified using the Cell Magic Wand plug-in with Image J. We quantified between 20 to 50 cells per image, with at least three technical replicates and three independent experiments per treatment group.

### Proliferation and apoptosis

Proliferation was quantified by labeling cells for 2hr with 10μM of 5-ethynyl-2’-deoxyuridine (EdU; Invitrogen) at 12hr following alcohol exposure, followed by Click chemistry to detect EdU using Alexa-594-Azide (Invitrogen) diluted in 100 mM CuSO_4_/100 mM ascorbic acid in Tris-buffered saline. Apoptosis was visualized using Alexa-594-conjugated Annexin-V (Abcam, Waltham MA), or the commercial kits TUNEL-TMR (Roche; Penzburg, Germany) and TUNEL-FITC (Promega, Madison WI) as per manufacturer’s recommendations. Nuclei were visualized using DAPI.

### Assessment of pre-rRNA content

Pre-rRNA was quantified from O9-1 cells or dissected chick crania at experimentally determined times following exposure to 0-100mM alcohol. Total RNA was isolated using Trizol reagent (Invitrogen) and cDNA synthesized exactly as previously described [41]. qPCR was performed using the SYBR Select Master Mix (ABI, #4472913) and Real-Time PCR system (Bio-Rad CFX96) and adhering to MIQE guidelines. Primers directed against the internal transcribed spacer-1 (ITS-1) and the internal normalization control β2 microglobulin for mouse and chick are presented in **S1 Table** and were obtained from IDT (Coralville IA). The 2^-ΔΔCT^ method was used to quantify relative RNA abundance.

Endogenous rRNA synthesis in individual cells was visualized using 5-ethynyluridine (EU; Invitrogen). O9-1 and chick primary NCCs were incubated with 80mM alcohol for 30min and then incubated with 100μM EU for an additional 30min before washing and fixation. Incorporated EU was visualized using Click chemistry and Alexa-594-Azide (Invitrogen) as above. Nuclei were visualized using DAPI.

### Cellular transfections

Expression vector plasmids for dominant-negative p53 (XE150DN P53-pCS2P+, #17033; AddGene, Watertown MA) and MDM2 (pCMV MDM2, #16441, AddGene) were grown in *E*.*coli* strain DH5α and purified using cesium chloride ultracentrifugation. O9-1 cells at 60–70% confluence were transfected with 3.5 μg plasmid diluted in 2ml Lipofectamine 3000 (Invitrogen) following the manufacturer’s recommendations. Cells were exposed to alcohol 6hr thereafter.

### Western blot analysis

O9-1 cells were lysed in buffer (50 mM Tris-HCl, pH 7.5, 150 mM NaCl, 0.5% NonidetP-40, 50 mM NaF, 1 mM NaVO_3_, 1 mM dithiothreitol, 1 mM phenylmethylsulfonyl fluoride). Total proteins were separated on a 10% SDS-PAGE reducing gel and transferred onto a PVDF membrane using the Trans-Blot Turbo Transfer System (#1704150, Bio-Rad, Hercules, California). P53 protein was quantified using the primary antibody #ab26 (1:500, Abcam) and normalized against GAPDH content (1:2000, G8795; Sigma). Both were detected using isotype-specific goat anti-mouse secondary antibodies (#1030–05, #1021–05; both 1:5000 from Southern Biotech) coupled to horseradish peroxidase, and visualized using the Radiance Q detection kit (#AC2101, Azure Biosystems, Dublin, CA) and chemiluminescence.

### Zebrafish transfections

Fertile eggs were injected at the 1–2 cell stage with morpholinos directed against p53, RPS3A, RPL5A, RPL11, NOLC1, and MDM2 (GeneTools; Philomath OR) and were immediately reincubated. A list of these morpholino sequences, and the concentrations used are presented in **S1 Table**. Transfected embryos were then treated with alcohol at 70% epiboly as described above.

### Statistical analysis

Data were checked for normalcy and analyzed with the appropriate statistical test (SigmaPlot 14; Systat Software, San Jose CA). Unpaired two-tailed T-tests were used when two groups were compared, and ANOVA when more than two groups were involved. *P*<0.05 was the level of significance. Results are mean ± SEM unless otherwise indicated.

## Results

### Acute alcohol exposure induces nucleolar stress in CNCs

In the whole transcriptome sequencing of early neural head folds following alcohol-exposure [35], the most significantly affected KEGG pathway was ribosome (KEGG #04150), and alcohol suppressed their abundance by 20–70%. Because the *G*. *gallus* transcriptome is incompletely annotated with respect to KEGG, we curated this dataset manually to investigate alcohol’s impact upon additional genes related to ribosome activity. This revealed 219 genes related to RBG that have significantly altered representation in alcohol-exposed neural folds (**S2 Table**). This included 13 genes involved in rRNA synthesis, 62 in rRNA processing and assembly, 27 in cytosolic and ribosomal tRNA synthesis, and 106 ribosomal proteins (66 nuclear, 40 mitochondria; **Table 1**). The vast preponderance of these genes were significantly suppressed by alcohol exposure. These included transcripts encoding multiple subunits of RNA polymerase I and III, tRNA synthetases, nearly all cytosolic and mitochondrial ribosome proteins, and enzymes and proteins that regulate rRNA processing and ribonucleotide modification including DEAD-box DNA helicases and pseudouridylate synthases. For a subset of these alcohol-suppressed genes (N = 28), cross-reference against online databases revealed that loss-of-function mutations in their human orthologs are linked to ribosomopathies and many of these are typified by craniofacial deficits (**Table 2**). Among these are genes linked to Treacher-Collins syndrome (*POLR1C*, *POLR1D*, *TCOF*), acrofacial dysostosis (*POLR1A*), Bowen-Conradi syndrome (*EMG1*), Wilson-Turner syndrome (*NOL9*), cerebrocostomandibular syndrome (*SNRPB*), and Diamond-Blackfan anemia (DBA; *RPL5*, *RPL11*, *RPL15*, *RPL26*, *RPL27*, *RPL35A*, *RPS10*, *RPS15A*, *RPS17*, *RPS26*, *RPS28*). Notably, these syndromes can be associated with facial deficits having shared similarities with those of FASD, including mandibular hypoplasia, epicanthal folds, flattened philtrum, and thin upper lip. These expression data suggested the hypothesis that alcohol exposure might suppress ribosome biogenesis within the CNC.

**Table 1:** Alcohol-responsive genes related to ribosome biogenesis in early neural folds*.

KEGG Process	# Genes Affected	Gene Numbers	Alcohol-Responsive Genes
rRNA Synthesis (#03020)	13	4 increased	EIF4ENIF1, POLR1A, POLR3A, RPS6KA6
		9 decreased	EIF4E2, EIF4EBP1, POLR1C, POLR1D, POLR1F, POLR3F, POLR3H, POLR3K, RPS6KB2
rRNA Processing & Assembly (#03008)	62	8 increased	DDX6, DDX17, LARP, PUS10, RANBP2, RANBP17, XOP1, XRN1
		53 decreased	CSNK2A1, DDX19B, DDX25, DDX27, DDX41, DDX52, DDX54, DKC1, EIF3J, EMG1, FCF1, FTSJ3, GAR1, GNL1, GNL3, GTPBP4, IMP4, LOC100857591, LSG1, MKI67IP, NOB1, NOL7, NOL9 (LAS1L), NOL12, NOP9, NOP16, NOP56, NOP58, NPM1, NPM3, NSUN4, NSUN5, PES1, PNO1, POP5, RAN, REXO2, RPF2, RPUSD4, RRNAD1, RRP7A, RRP8, RRP9, RRP12, TBL3, TCOF1, TSR1, UTP3, UTP4 (CIRH1A), UTP6, UTP11L, UTP14A, UTP23, WDR43
tRNAs (#00970)	16	1 increased	SEPSECS
		15 decreased	EARS2, FARSA, HARS, IARS2, KARS, MARS, NARS2, PARS2, QARS, RARS, SARS, SARS2, TARS2, YARS
tRNA Synthesis (#00970)	11	2 increased	TRMT11, XPOT
		9 decreased	AARSD1, AIMP1, AIMP2, DUS3L, GATC, MTO1, POP5, PSTK, PTRH2, PTRHD1
Nuclear Ribosomal Proteins (#04150)	66	0 increased	none
		66 decreased	RPL3, RPL4, RPL5, RPL6, RPL7, RPL7A, RPL8, RPL9, RPL10L, RPL10A, RPL11, RPL12, RPL13, RPL14, RPL15, RPL17L, RPL18A, RPL19, RPL21, RPL22, RPL22L1, RPL23, RPL23A, RPL24, RPL26, RPL27, RPL27A, RPL29, RPL30, RPL31, RPL32, RPL34L, RPL35, RPL35A, RPL36, RPL36A, RPL37, RPL37A, RPL38, RPL39, RPLP0, RPLP1, RPLP2, RPS2, RPS3, RPS3A, RPS4, RPS6, RPS8, RPS10, RPS11, RPS12, RPS13, RPS14, RPS15, RPS15A, RPS16, RPS17, RPS20, RPS23, RPS24, RPS25, RPS26, RPS27A, RPS28, RPS29, RPSA
Mitochondrial Ribosomal Proteins (#04150)	40	0 increased	none
		40 decreased	MRPL2, MRPL9, MRPL16, MRPL17, MRPL18, MRPL19, MRPL20, MRPL23, MRPL24, MRPL28, MRPL30, MRPL35, MRPL37, MRPL38, MRPL39, MRPL40, MRPL41, MRPL44, MRPL46, MRPL47, MRPL48, MRPL50, MRPL51, MRPL54, MRPL55, MRPS2, MRPS5, MRPS7, MRPS10, MRPS11, MRPS12, MRPS14, MRPS15, MRPS18C, MRPS21, MRPS22, MRPS23, MRPS25, MRPS26, MRPS34
p53 Signaling	25	12 increased	ATM, BNIP2, CCND2, MDM2, MDM4, PAK2, PAK3, RASA1, RASAL2, RRM2B, TP53I11, TP53INP1
		13 decreased	BAG4, BCL2L1, BCL7B, CCNB2, CCNB3, CNDBP1, CDK6, GNL3, PAK4, RASA4, RPS19BP1, SIVA1, TP53RK

* Fold-changes, P-adjusted values, and Ensemble IDs for these genes are presented in S1 Table.

**Table 2:** Alcohol-suppressed genes linked to human ribosomopathies*.

Disorder	Craniofacial Deficits?	Gene ID	Gene	FC Alc/Con	P-adj	Wikigene Description
5q-syndrome	N	ENSGALG00000004588	RPS14	0.666	7.70E-06	Ribosomal protein S14
Acrofacial dysostosis	Y	ENSGALG00000015765	POLR1A	1.250	8.26E-02	Polymerase (RNA) I polypeptide A
Bowen-Conradi	Y	ENSGALG00000014568	EMG1	0.669	8.49E-04	EMG1 nucleolar protein homolog
Diamond-Blackfan Anemia	Y	ENSGALG00000005922	RPL5	0.759	3.75E-03	Ribosomal protein L5
	?	ENSGALG00000000150	RPL9	0.683	3.23E-05	Ribosomal protein L9
	Y	ENSGALG00000003971	RPL11	0.694	7.52E-02	Ribosomal protein L11
	Y	ENSGALG00000011290	RPL15	0.619	6.59E-08	Ribosomal protein L15
	Y	ENSGALG00000002868	RPL26	0.614	4.26E-08	Ribosomal protein L26-like
	Y	ENSGALG00000002837	RPL27	0.588	1.71E-09	Ribosomal protein L27
	?	ENSGALG00000016775	RPL31	0.604	1.41E-08	Ribosomal protein L31
	N	ENSGALG00000001039	RPL35	0.512	1.00E-02	Ribosomal protein L35
	Y	ENSGALG00000007611	RPL35A	0.582	2.08E-05	Ribosomal protein L35a
	Y	ENSGALG00000002813	RPS10	0.591	2.23E-09	Ribosomal protein S10
	Y	ENSGALG00000006771	RPS15A	0.588	1.91E-02	Ribosomal protein S15a
	Y	ENSGALG00000002157	RPS17	0.372	1.59E-12	Ribosomal protein S17
	?	ENSGALG00000004871	RPS24	0.617	4.10E-04	Ribosomal protein S24
	Y	ENSGALG00000027807	RPS26	0.561	4.50E-04	Ribosomal protein S26
	Y	ENSGALG00000024398	RPS28	0.511	3.49E-11	Ribosomal protein S28
	N	ENSGALG00000012229	RPS29	0.568	8.67E-08	Ribosomal protein S29
	Y	ENSGALG00000010077	RPS3A	0.708	1.52E-04	Ribosomal protein S3A
Dyskeratosis Congenita	N	ENSGALG00000005054	DKC1	0.826	7.46E-02	Dyskerin
	N	ENSGALG00000024261	NHP2	0.510	3.66E-11	NHP2 ribonucleoprotein homolog
RPS23-related	N	ENSGALG00000001634	RPL23	0.558	5.32E-07	Ribosomal protein L23
Treacher Collins	Y	ENSGALG00000010349	POLR1C	0.716	1.84E-03	Polymerase (RNA) I polypeptide C
	Y	ENSGALG00000007470	POLR1D	0.685	9.74E-03	Polymerase (RNA) I polypeptide D
	Y	ENSGALG00000005535	TCOF1	0.727	1.07E-03	Treacher Collins-Franceschetti Synd. 1
Widemann-Rautenstrauch	N	ENSGALG00000004947	POLR3A	1.362	4.13E-02	Polymerase (RNA) III (DNA directed) polypeptide A, 155kDa
X-Linked	Y	ENSGALG00000027227	RPL10	0.495	3.54E-15	Ribosomal protein L10
Wilson-Turner	Y	ENSGALG00000028958	NOL9	0.734	1.82E-03	Nucleolar protein 9

* Ribosomopathy and craniofacial information regarding the human orthologs of these alcohol-responsive genes are from the online databases Online Mendelian Inheritance in Man (www.OMIM.org), MalaCards (www.malacards.org), GeneCards (www.genecards.org), and PubMed. FC, fold change. Y is yes, N is no, ‘?’ indicates unclear effect.

**Fig 1:**
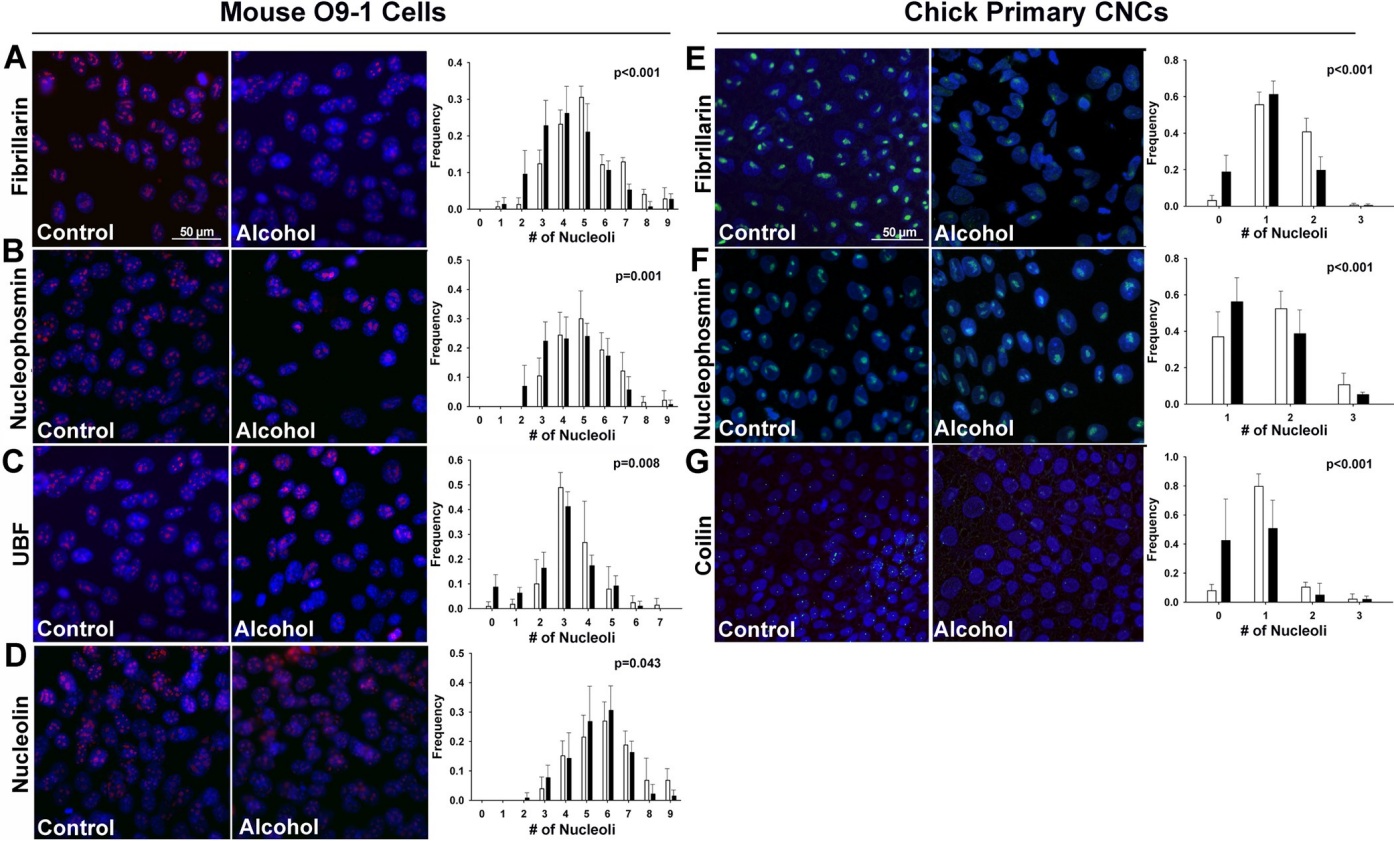
Acute alcohol exposure causes nucleolar stress in the mouse CNC line O9-1 and in primary chick CNCs. **(A-D)** Murine O9-1 cells were exposed to 60mM ethanol for 2hr and assessed 6hr after the alcohol addition. Compared to untreated cells (Control, left panels), alcohol (middle panel) altered the distribution of nucleoli per nucleus such that there were fewer nucleoli in alcohol-exposed CNCs, as assessed by immunostain for the nucleolar proteins **(A)** fibrillarin, **(B)** nucleophosmin, **(C)** UBF, and **(D)** nucleolin. Values that quantify the distribution of nucleolar number per nucleus (right panels) are the mean ± SD of three independent experiments as detailed in *Methods*. **(E-G)** Primary chick CNCs, derived from cranial explants, were exposed to 60mM ethanol for 2hr and assessed 6hr after the alcohol addition. Compared to untreated cells (left panels), alcohol (middle panels) altered the distribution of nucleoli per nucleus such that there were fewer nucleoli in alcohol-exposed CNCs, as visualized using immunostain for **(E)** fibrillarin, **(F)** nucleophosmin, and **(G)** coilin. Values that quantify the distribution of nucleolar number per nucleus (right panels) are the mean ± SD for N = 3–7 experimental replicates as detailed in *Methods*. For all, nuclei were imaged using DAPI counterstain and representative images are shown; scale bar represents 50 μm. Data were analyzed using chi-square analysis.

Using antibodies directed against components of the nucleolus, we explored alcohol’s potential impact upon nucleolar stress responses in the CNC line, O9-1, quantifying the number of nucleolar nodes, or sites of RBG per nucleus; this is an accepted metric for nucleolar stress [42, 43]. Murine nuclei contain as many as 9 discernable nucleoli, and acute alcohol exposure (60 mM, 6 hr) altered the distribution of the number of nucleolar structures such that more alcohol-exposed CNCs had 0 to 3 nucleoli and fewer had 7–9 nucleoli as compared with unexposed CNCs (**Fig 1A–1D**). This shifted distribution toward fewer nucleoli was observed for multiple components that comprise the nucleolar structure, as visualized for fibrillarin, which is part of the box C/D snoRNP and catalyzes the methylation of 2′-hydroxy-ribose in pre-rRNA (p<0.001; Fig 1A); nucleophosmin, which associates with the ribonucleoprotein complex and may assist nucleolar protein transport (p = 0.001; Fig 1B); the upstream binding factor (UBF), which contributes to rRNA transcription (; p = 0.008; Fig 1C); and nucleolin, which associates with nucleophosmin and contributes to ribosome assembly (p = 0.043; Fig 1D; all values mean ± SEM).

We corroborated these cell line findings in primary CNCs. Up to three nucleoli per nucleus are readily visualized in avian cells as sites of pre-rRNA processing. Similar to findings in the O9-1 cells, primary CNCs exposed to alcohol (60 mM, 6 hr) had a significantly altered distribution of nucleoli per nucleus and had fewer nucleolar nodes (**Fig 1E–1G**) as visualized using antibodies directed against fibrillarin (p<0.001; Fig 1E), nucleophosmin (p<0.001; Fig 1F; all means ± SEM), and coilin, a component of Cajal bodies that contributes to the post-transcriptional modification of small nucleolar RNAs (p<0.001; Fig 1G). Of the genes encoding these nucleolar proteins, only *Npm1* (nucleophosmin) had reduced expression in response to alcohol (76.2% of control, Padj = 4.16E-03, **S1 Table**), indicating that reduced expression did not account for these nucleolar reductions. Thus, in two different CNC models from disparate species, and using an array of markers that identify multiple steps of ribosome biogenesis, acute alcohol exposure induced the dissolution of nucleolar structures, consistent with nucleolar stress, in CNC progenitors.

### Dose-response and time course

**Fig 2:**
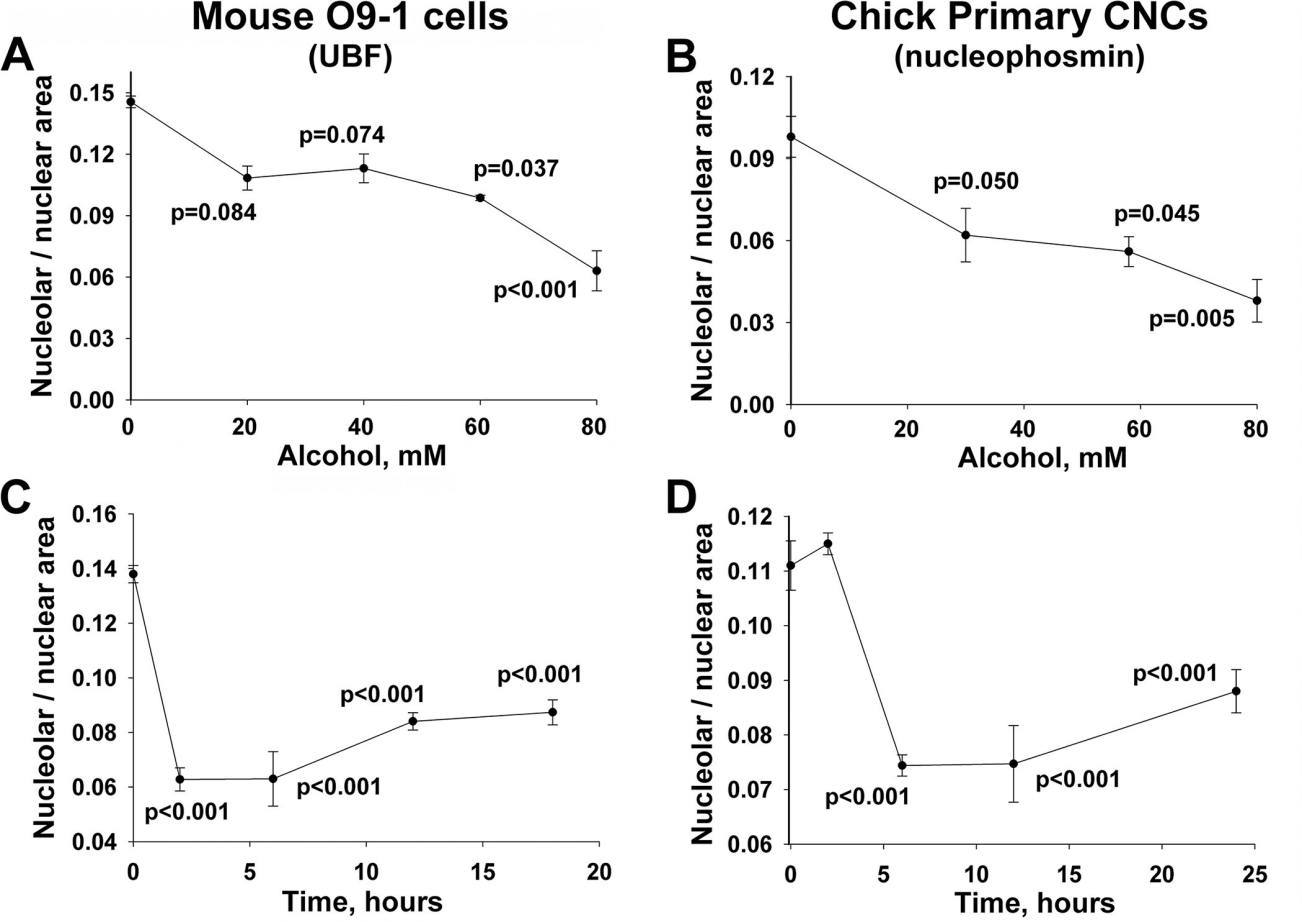
Alcohol-induced nucleolar stress in mouse and chick CNCs is dose-dependent and persistent. **(A, B)** Dose-dependence of nucleolar stress. Alcohol reduced the nucleolar area per nucleus in a dose-dependent manner, at concentrations as low as 60mM for O9-1 cells **(**for UBF; **A)** and 29mM for primary CNCs **(**for nucleophosmin; **B)**. Values are the mean ± SEM from 4 (O9-1) and 8 (primary CNCs) independent experiments. **(C, D)** Timecourse of nucleolar stress. The nucleolar reduction is rapid and occurs within 2hr (O9-1; 80mM) and 6hr (primary CNCs; 60mM) following the addition of alcohol at each cell lineage’s calculated EC50. Moreover, the nucleolar abundance remains low in both populations for at least 24hr following the 2hr alcohol exposure. Values are mean ± SEM from 12 (O9-1) and 8 (primary CNCs) independent experiments. For all analyses, nucleoli were visualized using antibodies directed against UBF (O9-1) or nucleophosmin (primary CNCs), and nuclei were visualized using DAPI. Data were analyzed using one-way analysis of variance with Holm-Sidak multiple comparisons.

The alcohol concentrations that induced nucleolar stress were pharmacologically relevant, were similar in both CNC models, and had similar slopes. Alcohol (60 mM, 2hr) reduced the nuclear area occupied by UBF (CON, 0.146 ± 0.009, ALC, 0.099 ± 0.003; p<0.01; **Fig 2A**) and nucleophosmin (CON, 0.097 ± 0.004, ALC, 0.075 ± 0.003; p = 0.009; all means ± SEM; **Fig 2B**) in O9-1 and primary CNC, respectively. For both CNC lineages, alcohol concentrations as low as 20 to 30 mM reduced the nucleolar area, although this was a trend at lower alcohol doses in the O9-1 cells (p≤0.084) and significant for primary CNCs (p≤0.050); for comparison, a blood alcohol concentration of 0.08mg% corresponds to 17 mM ethanol. The calculated EC50 for alcohol’s effect, based on linear regression to 0 mM, was 78 mM for O9-1 cells and 63 mM for primary CNCs. At the EC50, alcohol’s invocation of nucleolar stress was rapid. For O9-1 cells the nucleolar dissolution occurred within 2hr of alcohol addition (**Fig 2C**), and for primary CNCs occurred between 2hr and 5hr following alcohol exposure (**Fig 2D**). Moreover, this nucleolar suppression lasted for at least 18 hr to 24 hr in both CNC lineages, indicating that the nuclear stress persisted well beyond the period of direct alcohol exposure. Thus, alcohol’s induction of nucleolar stress was rapid, persistent, and occurred at pharmacologically relevant alcohol concentrations.

### Alcohol suppresses rRNA synthesis in primary CNCs

**Fig 3:**
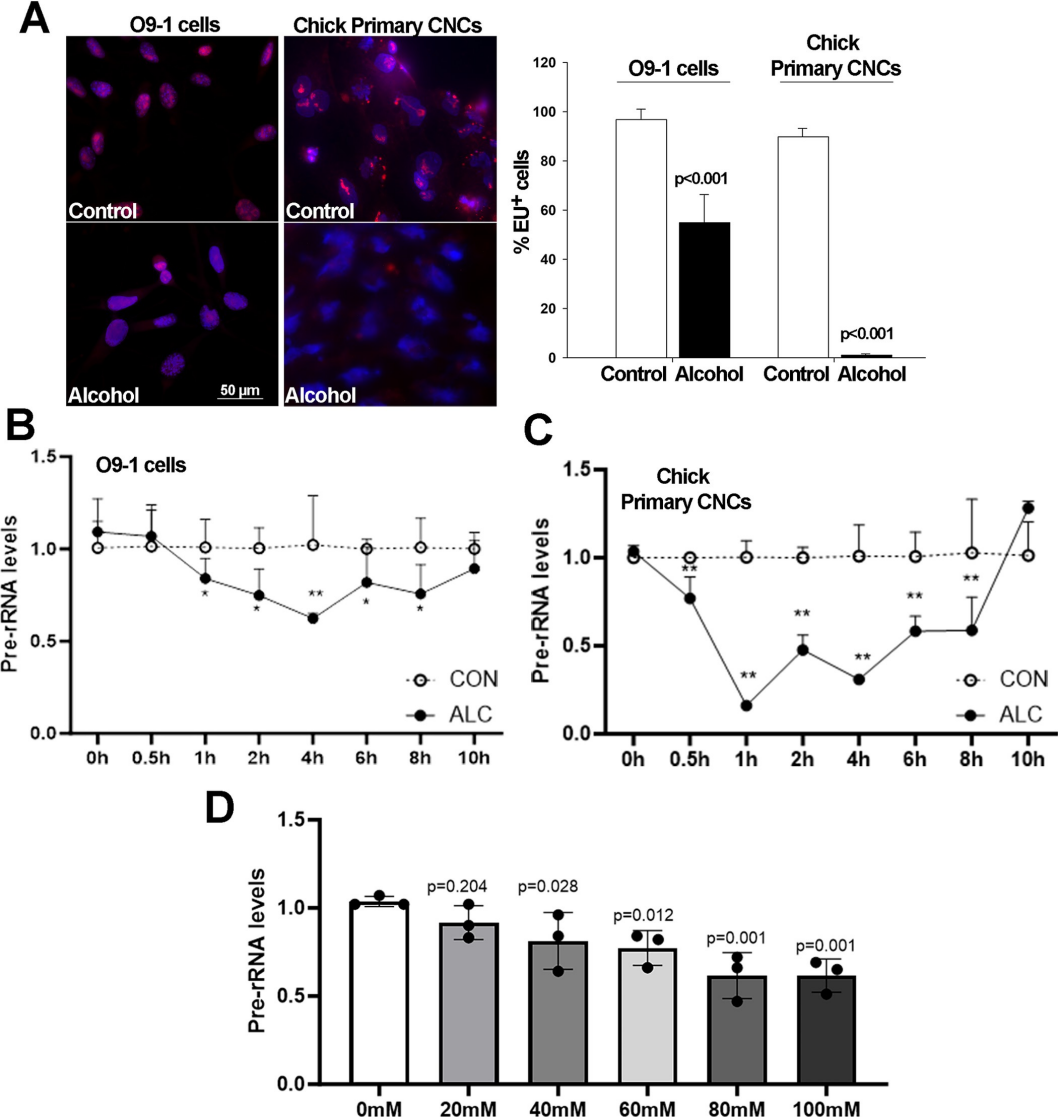
Alcohol suppresses *de novo* rRNA synthesis and content in O9-1 cells and primary chick CNCs. **(A)** Alcohol exposure at the EC50 for O9-1 cells (80mM) and chick primary CNCs (52mM) reveals fewer or no EU-positive nuclei (red) in alcohol-exposed nuclei (blue) as compared with controls. Nuclei visualized using DAPI; bar indicates 50 μm. (B) Dose-response shows a significant decline in *de novo* rRNA synthesis, quantified using qPCR for ITS-1, at alcohol exposures at or above 40mM for O9-1 cells. **(C)** At 30min following exposure to 80mM alcohol there is a significant decline in ITS-1 content in O9-1 cells. **(D)** At 30min following exposure to 52mM alcohol there is a significant decline in ITS-1 content in chick primary CNCs. Values are mean ± SD for three independent replicates. Data analyzed using two-tailed Student’s t-test (A) or one-way analysis of variance (B-D).

Nucleolar stress can be accompanied by the cessation of rRNA synthesis, and this loss of rRNA substrate can be an instigating factor for the dissolution of nucleolar protein interactions. We assessed *de novo* rRNA synthesis in primary CNCs using the rUTP analog 5-ethynyl uridine (EU), which is directly incorporated into RNA. In untreated CNCs, sites of EU incorporation indicating nucleolar synthesis of rRNA were colocalized within the nuclei (**Fig 3A**). At 1 hour following the addition of alcohol at the EC50 (80mM for O9-1, 52mM for primary CNCs), *de novo* rRNA synthesis was detected in only 54.9 ± 11.4% of O9-1 cells, and was not detected in primary CNCs (p<0.001 for both lineages). Using qPCR to detect the internal transcribed spacer-1 (ITS-1), which is located between the 18S and 5.8S rRNA genes and is lost during rRNA processing, we found a rapid, alcohol-mediated decline in newly synthesized rRNA. Pre-rRNA levels were reduced within 1hr of exposure to the EC50 dose for O9-1 cells (**Fig 3B**), and within 30min of exposure to the EC50 dose for chick primary CNCs (**Fig 3C**). The reduction was stronger in the primary CNCs, and for both lineages the decline was persistent and were not normalized until 10hr following the alcohol exposure; this persistence is notable as alcohol is volatile and its levels in the culture media are <9mM by 2hr following the initial exposure [32]. Reductions in pre-rRNA content were detected at alcohol exposures of 40mM and below (**Fig 3D**).

### Alcohol-induced nucleolar stress causes CNC apoptosis via p53

**Fig 4:**
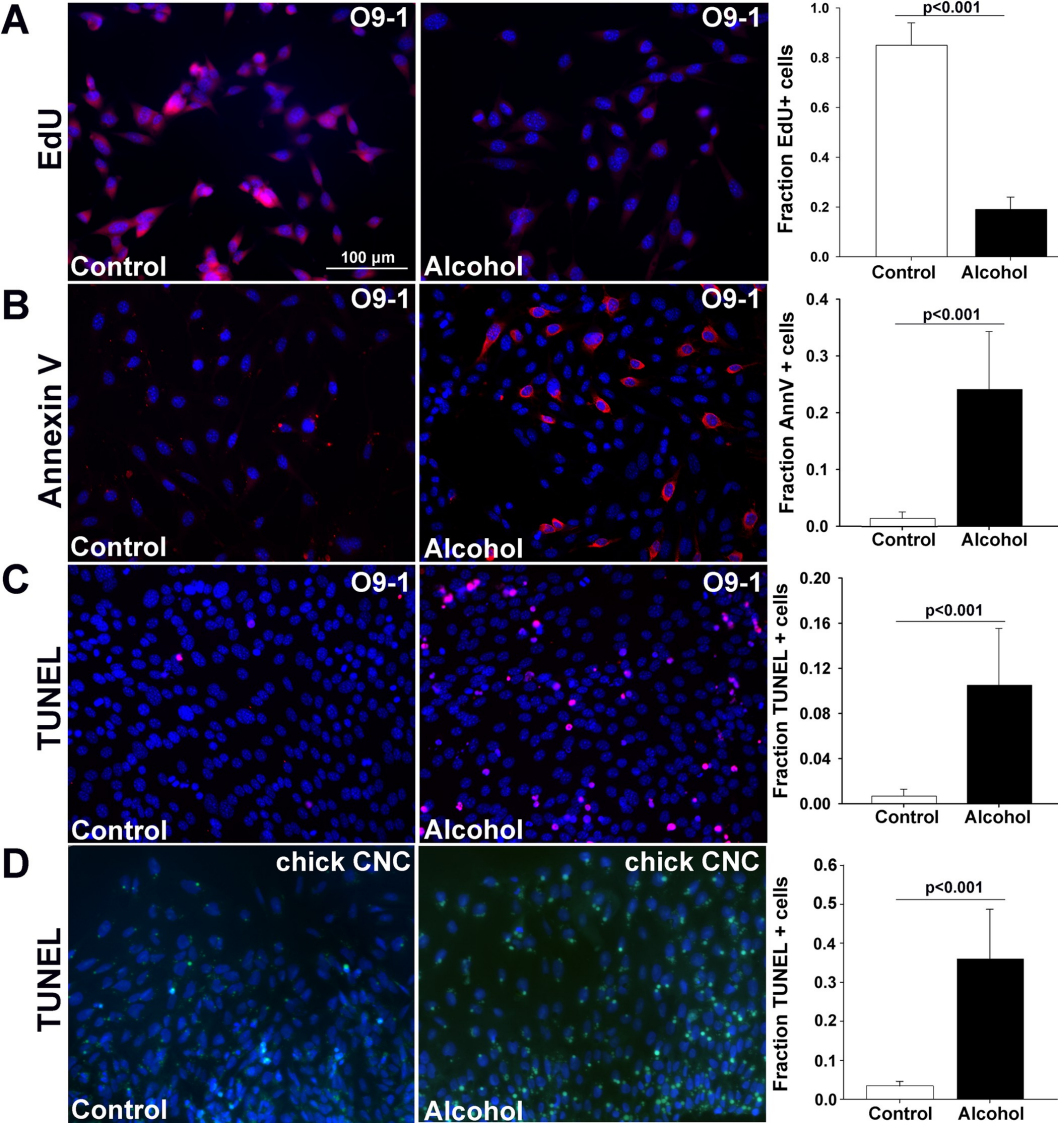
Alcohol causes apoptosis in the O9-1 cells and primary chick CNCs. Cells were exposed to alcohol at the EC50 (80mM for O9-1 cells, 52mM for primary CNCs) for 2hr and assessed at times thereafter. **(A)** Alcohol reduced the percentage of EdU+ proliferating cells in O9-1 cells 12hr after exposure. **(B)** Alcohol increased the percentage of Annexin-V^+^ (red) cells in O9-1 cells 6hr after exposure. **(C)** Alcohol increased the percentage of TUNEL^+^ (red) O9-1 cells 18hr after exposure. **(D)** Alcohol increased the percentage of TUNEL^+^ (green) primary chick CNCs at 18hr after exposure. Values are the mean ± SEM of 5–8 independent experiments. Data were analyzed using two-tailed Student’s t-test.

Ribosome dysbiogenesis and nucleolar stress cause the cessation of proliferation and the apoptotic elimination of primary CNCs in the early embryo via p53-mediated mechanisms [44]. Alcohol exposure caused a 4.5-fold reduction in the proliferation of O9-1 cells as assessed using EdU incorporation at 12hr after exposure (CON, 85.9% ± 4.2%, ALC, 18.79% ± 2.4%; p<0.001; **Fig 4A**). Alcohol exposure also causes apoptosis in these primary CNCs and this also requires p53 [32–34], but whether similar vulnerability exists in the murine O9-1 cells is unknown. Alcohol exposure at the EC50 (80 mM, 2hr) induced significant apoptosis in the O9-1 cells by 16hr following the exposure, as assessed using both immunostaining for Annexin V (CON, 1.5% ± 0.4%, ALC, 22.8% ± 10.0%; p<0.001; **Fig 4B**) and in situ DNA end-labeling (i.e., TUNEL; CON, 0.6% ± 0.6%, ALC, 9.7% ± 4.7%; p<0.001; **Fig 4C**). This is consistent with the well-documented apoptosis observed in alcohol-exposed primary CNCs (CON, 3.4.5% ± 1.2%, ALC, 36.0% ± 12.7%; p<0.001; **Fig 4D**) and confirms that alcohol exposures that induce nucleolar stress in O9-1 cells are also pro-apoptotic.

**Fig 5:**
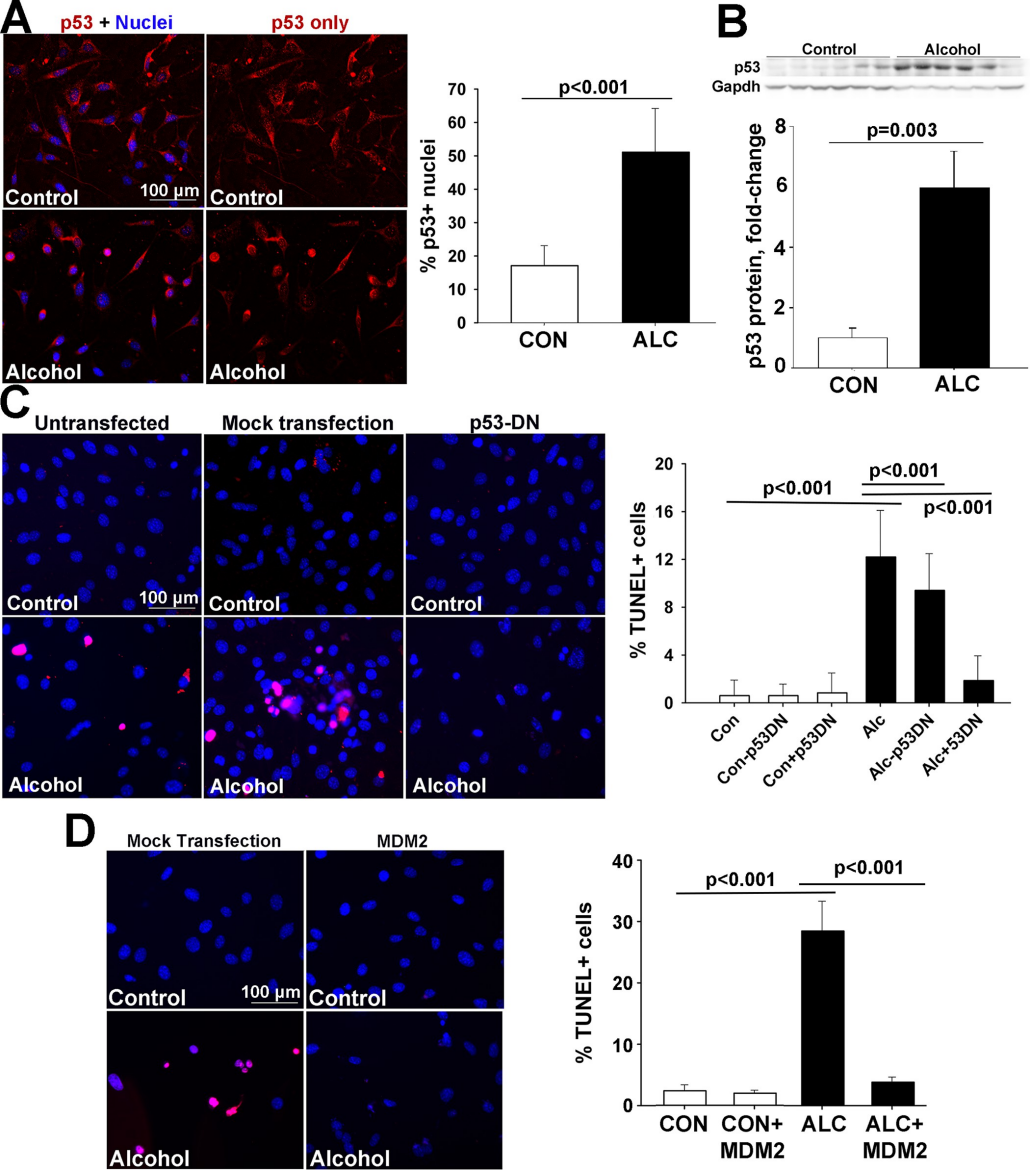
Alcohol causes p53/MDM2-dependent apoptosis in the mouse CNC line O9-1. **(A)** Immunostain reveals that p53 protein (red) is detected predominantly in the cytosol of untreated O9-1 cells, and this protein becomes stabilized within the nuclei (blue) 6hr after alcohol exposure. Right panel, alcohol increases the percentage of p53^+^ nuclei. **(B)** Alcohol exposure causes a six-fold rise in p53 protein content in O9-1 cells at 6hr following exposure. **(C)** Transfection of dominant-negative p53 (p53-DN) into O9-1 cells prevents the alcohol-induced apoptosis as detected using TUNEL (red signal) and does not affect the survival of unexposed cells, whereas transfection with the parent pCS2 vector does not affect cell survival. Right panel, alcohol increases the percentage of TUNEL^+^ cells and this is prevented by overexpression of dominant-negative p53 (+p53DN) but not by transfection with the empty vector (-p53DN). **(D)** Overexpression of MDM2 prevents the alcohol-induced apoptosis as detected by TUNEL (red) but does not affect the survival of control cells, compared with cells transfected with the empty vector pCS2. Right panel, alcohol increases the percentage of TUNEL+ O9-1 cells and this is normalized by MDM2. All values are mean ± SEM of 2 independent experiments as detailed in *Methods*. Data were analyzed using equal variance t-test (A, B) or one-way analysis of variance with Holm-Sidak multiple comparisons (C, D).

Nucleolar stress alters cellular activity and fate through its activation of the transcriptional effector p53, which can then promote apoptosis. Primary CNCs express p53 and under low-stress conditions the protein is predominantly cytosolic (**Fig 5A)** [23, 32, 45]. Immunostain revealed that alcohol exposures that induced nucleolar stress also led to the stabilization of nuclear p53 (CON, 17.1 ± 2.3%, ALC, 51.1 ± 4.9% p53+ nuclei; p<0.001) and might be accompanied by a reduction in its cytosolic content **(Fig 5A**). Western blot analysis confirmed that this stabilization reflected a six-fold increase of p53 protein content in alcohol-exposed cells (**Fig 5B**). That this p53 is mechanistic in their alcohol-induced apoptosis was evidenced through overexpression of a dominant-negative p53 (p53-DN) variant, which prevented apoptosis within alcohol-exposed cells as measured by TUNEL, and did not affect the frequency of apoptosis in unexposed cells (CON, 0.6 ± 0.5%, ALC, 12.2 ± 2.0%, CON + p53-DN, 0.8 ± 0.8%, ALC + p53-DN, 1.9 ± 0.7% TUNEL+ nuclei; p<0.001; **Fig 5C**).

In many cell lineages including CNCs, interactions with MDM2 destabilize p53 and target it for proteasomal degradation [18]. Under nucleolar stress, select ribosomal proteins interact with MDM2, displacing p53 and thus enable p53 to act as a transcriptional effector [46]. Overexpression of MDM2 prevented the apoptosis of alcohol-exposed O9-1 cells, as assessed by TUNEL, and did not further affect cell survival in controls (CON, 2.4 ± 1.0%, ALC, 28.5 ± 4.8%; CON+MDM2, 2.0 ± 0.5%, ALC+MDM2, 3.8 ± 0.9% TUNEL+ nuclei; p≤0.001; **Fig 5C**).

### Ribosome dysbiogenesis contributes to the alcohol craniofacial dysmorphology

**Fig 6:**
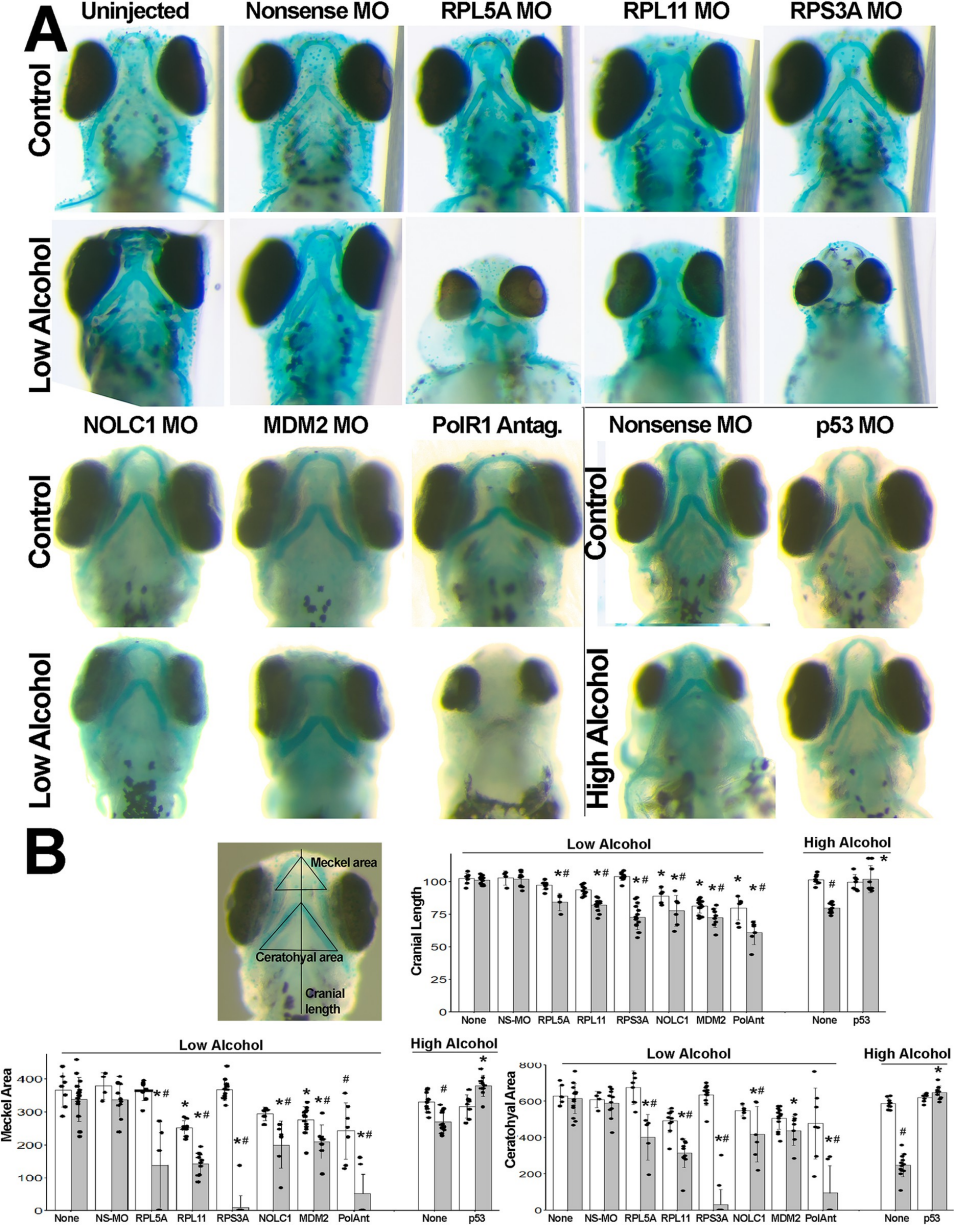
Haploinsufficiency in ribosome biogenesis-related genes heightens vulnerability to alcohol-induced craniofacial deficits in zebrafish embryos. **(A)** Zebrafish embryos injected with nonsense MO, or MOs against *RPL5A*, *RPL11*, *RPS3A*, *NOLC1*, *p53*, or *MDM2* had no or modest deficits in the size or shape of cranial cartilage elements as compared with no-MO controls. Low-dose alcohol-only also did not worsen cranial development. The combination of low-dose alcohol and morpholinos directed against genes that promote ribosome biogenesis (*rpl5a*, *rpl11*, *rps3a*, *nolc1*) and p53/MDM2 signaling (*mdm2*) synergized to worsen cranial development and, in some instances, ablated cranial cartilage. Similarly, low-doses of the RNA polymerase I inhibitor CX5461 modestly affected cranial cartilage and synergized with low-dose alcohol to eliminate facial cartilage. Conversely, high-dose alcohol (boxed panels) caused craniofacial reductions that were prevented by morpholinos directed against p53. All views are ventral at 4 dpf with equivalent magnification. **(B)** Depiction of morphometry measurements, and quantification of cranial length, Meckel area, and ceratohyal area. Units are arbitrary and normalized to the mean of the uninjected controls. Open bars, no-alcohol; shaded bars, alcohol at the low dose or high dose as indicated. All values are mean ± SD with 4–14 embryos per treatment as detailed in Methods. Data analyzed using two-way analysis of variance followed by post hoc analysis using Holm-Sidak multiple comparisons (morpholino, compound) or within-group comparisons (alcohol vs. no-alcohol). * Different at p≤0.001 from its exposure-matched control for either nonsense-MO vs. gene-morpholino or no-compound vs. compound-treated. # Different at p≤0.001 from its treatment-matched control, Alcohol vs. no-Alcohol.

We postulated that, if the loss of RBG was mechanistically involved with the alcohol-induced CNC losses and craniofacial dysmorphology, then insufficiency in the expression of genes that participate in RBG would sensitize CNCs to alcohol and heighten their vulnerability to alcohol-induced craniofacial deficits. We tested this using an established approach [35] and our established zebrafish model [31], administering lower concentrations of morpholinos and alcohol that separately cause modest facial deficits, and testing their ability when co-administered to synergize and exacerbate those deficits. We tested five genes that are alcohol responsive in primary CNCs (Table 1) and contribute to craniofacial deficits in the ribosomopathies Diamond-Blackfan anemia (*Rpl5*, *Rpl11*, *Rps3a*), Treacher-Collins Syndrome (*Nolc1*) and p53-mediated apoptosis (*Mdm2*) [5, 19, 20, 23]. As assessed both visually (**Fig 6A**) and by quantifying cranial elements derived from CNCs (**Fig 6B**), we found significant effects of exposure status (F_(1,125)_ = 118.1 / 201.5 / 150.2, p<0.001), gene target (F_(1,125)_ = 45.6 / 30.3 / 26.9, p<0.001) and exposure × gene target (F_(1, 125)_ = 12.0 / 22.1 / 24.0, p<0.001) upon cranial length, Meckel cartilage area, and ceratohyoid area, respectively. Morpholinos having a nonsense sequence did not affect cranial development in control or alcohol-exposed embryos. In embryos that did not receive alcohol, morpholinos directed against *Rpl5a* and *Rps3a* did not affect facial cartilage or outgrowth; those directed against *Mdm2* (p<0.001) and *Nolc1* (p = 0.019) modestly reduced cranial length, and those targeting *Mdm2* (p = 0.003) and *Rpl11* (p = 0.01) reduced the area bounded by Meckel’s cartilage. Although this moderate alcohol dose did not affect the facial cartilage or outgrowth in the absence of morpholinos or with the nonsense morpholino, the combination of alcohol and targeting morpholinos significantly impaired craniofacial development. Specifically, alcohol plus morpholinos directed against *Rpl5a*, *Rpl11*, *Rps3a*, *Nolc1* and *Mdm2* reduced cranial length and either stunted or obliterated the formation of Meckel cartilage and the ceratohyal cartilage, whether compared against alcohol-only (p<0.001) or no alcohol + nonsense-morpholino controls (p<0.001). We complemented this with the small molecular antagonist CX-5461, which is an inhibitor of the RNA Polymerase 1 complex that transcribes rRNA [47]. We used a concentration of CX-5461 that generated a normal-looking cranial appearance with reduced cranial length and Meckel area (both p<0.001) in the no-alcohol controls. In combination with the moderate alcohol exposure, this same concentration caused strong cranial stunting with complete or near-complete ablation of the Meckel and ceratohyal cartilages (all p<0.001).

Conversely, we hypothesized that, if p53 contributed to the apoptosis of these alcohol-exposed CNCs, then morpholinos directed against p53 would attenuate the alcohol-associated craniofacial deficits. Here, we used a high-dose alcohol exposure (500mM in embryo media; final embryo concentration 180mM [31, 40]) that significantly reduced cranial length (F_(3,36)_ = 53.8, p<0.001) and the Meckel (F_(3,36)_ = 20.3, p<0.001) and ceratohyal areas (F_(3,36)_ = 193.8, p<0.001). The addition of morpholinos directed against p53 did not affect craniofacial development in control embryos. For embryos exposed to the high dose alcohol, the p53-directed morpholinos completely prevented the reductions in cranial length and the Meckel and ceratohyal areas (p<0.001) otherwise caused by the high-dose alcohol.

## Discussion

This is the first report that alcohol causes nucleolar stress in any cell lineage, and that this nucleolar stress has a mechanistic role in alcohol’s adverse effects upon cell growth and survival. Alcohol’s suppression of rRNA synthesis and of nucleolar stress initiates the p53-mediated apoptosis of CNC progenitors and contributes to the facial deficits that partly characterize PAE. This mechanism is relevant across three distinct groups–avians, teleost fish, and rodents–indicating it represents a conserved mechanism of alcohol’s action and thus may also be relevant in humans. Previously, ribosomopathies were predominantly understood as loss-of-function mutations in genes contributing to RBG (i.e., [1, 3, 5]. To our knowledge, this is the first report that extends those origins to an environmental exposure and specifically the human teratogen alcohol. Given that in the U.S. 5.2% of pregnant people self-report binge drinking (≥4 drinks/occasion) in the prior thirty days [48], the population that potentially experiences prenatal nucleolar stress may be considerable.

That pharmacologically relevant alcohol exposures cause nucleolar stress perhaps should not be surprising given the numerous hints within the alcohol-related literature. Sulik noted the facial similarities between fetal alcohol syndrome and the ribosomopathy Treacher-Collins syndrome decades ago [49], and several ribosomopathies including Treacher-Collins, Diamond-Blackfan, and Robert’s syndrome [3, 22] share diagnostic facial features with those for PAE including flattened/missing philtrum, thin upper lip, and epicanthal folds [28]. Molecular-level studies further suggest an association, as acute alcohol exposure rapidly suppresses the expression of ribosome proteins and related genes in the early neural fold [35], the O9-1 CNC lineage, mouse hepatocytes [50, 51], and mouse cortical neuroprogenitors [41]. In otherwise untreated chick strains having differential vulnerability to alcohol-induced CNC apoptosis, the KEGG pathway having the greatest altered representation was that for Ribosome and the reductions in individual RPs in the vulnerable strain ranged from 20–70% (p-adj = 10E-47) [36]. Reductions in mitochondrial RPs were noted in mouse hepatocytes [50]. Similarly, the ribosomal gene cluster was independently flagged as suppressed in expression studies of mouse headfolds in strains having differential alcohol vulnerability [52, 53], and in a meta-analysis of rodent gene array sets derived from alcohol-exposed embryos, whole brains, and liver and kidney [54]. In zebrafish, haploinsufficiency in methionine tRNA synthase (*mars*), essential for translation initiation, heightens CNC sensitivity to alcohol’s apoptosis and craniofacial deficits [55]. Ribosome proteins are repeatedly identified as suppressed under both acute and chronic alcohol exposure in non-embryonic tissues, changes attributed to non-specific stress responses and/or metabolic adaptations to chronic exposure [50, 51, 56, 57]. However, the functional relevance of that alcohol-driven repression in the context of nucleolar stress had not been noted in those reports.

Results are also consistent with demonstrations that alcohol activates p53 in multiple cell lineages [33, 58, 59]; however, the underlying mechanism responsible for its activation has remained elusive. Exposures similar to those used here stabilize nuclear p53 protein in primary avian- and mouse-derived CNCs [32–34]. This stabilized p53 is essential for their alcohol-mediated apoptosis, and both p53 haploinsufficiency or blockade with small molecule inhibitors confers resistance to that cell death and normalizes craniofacial outcome [32, 34]. The current work extends those studies to show that this p53 stabilization is preceded by nucleolar stress, and that MDM2 overexpression prevents this apoptosis and normalizes craniofacial development in the presence of alcohol. This involvement of RP/MDM2/P53 signaling in the alcohol-mediated nucleolar stress is consistent with genetic explorations of nucleolar stress in CNCs, in which loss-of-function in a range of RBG-related genes cause p53-mediated apoptosis including MDM2 itself [26], RNA pol I [25, 60], the rRNA processing protein TCOF [23, 61], the RBG promoter Wdr43 [62] and select ribosomal proteins known to interact with MDM2 [17, 19, 20, 63–65]. Here, the reduction through genetic or molecular means in several of these effectors including RNA Pol 1, TCOF (NOLC), and RPs heightens vulnerability to alcohol-driven craniofacial deficits, whereas those deficits are attenuated by effectors known to stabilize RBG including MDM2 itself. These studies expand our understanding of p53’s role in mediating alcohol-driven apoptosis.

It remains unclear why CNCs are vulnerable to ribosome dysbiogenesis while other proliferative embryonic lineages are not, especially given the global need for functioning ribosomes. One potential explanation is that some lineages rely on specialized ribosomes that have different functions, for example, the unique protein-folding abilities contributed by RPL39L in spermatocytes [66]; however, RPL39L is absent from CNCs [35]. Cells also can differ in the transcriptional effectors that drive ribosome-related gene expression, for example, deficits in GATA1 contribute to some forms of Diamond-Blackfan anemia, and these might respond differently to alcohol [67, 68]. Many lineages vulnerable to ribosomopathies have an elevated reliance on ribosomes, reflecting a need for replenishment under high proliferation rates (such as erythrocytes and enterocytes) or higher demands for protein synthesis per se (such as hair follicles) [69]. In such highly proliferative cells, the energy costs for ribosome replacement can occupy 70–80% of the cell’s total energy budget [7]. Premigratory CNCs have mitotic indices as high as 85% and doubling times from 16 to 24 hours [70], and their need to double their ribosome content with each cell division may enhance their vulnerability to ribosome dysbiogenesis and nucleolar stress.

An additional contributory factor may be related to the CNCs dual need to undergo both proliferative expansion and epithelial-mesenchymal transformation (EMT) prior to their migration into the facial anlage. P53 is enriched in premigratory CNCs and is reduced immediately prior their delamination [26]. Although p53’s role in CNC is unclear, evidence suggests it may serve as a rheostat to control this proliferative / migratory balance through regulation of the cell cycle and apoptosis. This is seen in the increased CNC proliferation and expanded numbers of PAX7^+^-SOX9^+^ CNCs under P53 loss-of-function [26], and their elevated apoptosis under nuclear p53 stabilization [5, 23]; this latter also suppresses the expression of Snai2 and Ets1, which initiate EMT [26]. The p53 protein in otherwise untreated O9-1 CNCs is predominantly cytosolic, similar to that observed in primary chick CNCs [32], and this subcellular compartmentalization could explain why these cells are proliferative despite the protein’s presence. The unique need of CNCs to regulate their competing choices of proliferation versus migration, and their use of p53 to do so, may underlie their heightened vulnerability to ribosomopathies. We speculate that alcohol’s suppression of rRNA synthesis and RBG initiates a state of nucleolar stress, which in turn signals an imbalance in their proliferative and/or migratory signals and thereby activates p53 to initiate cell cycle arrest and apoptosis. This may also explain why cortical neuronal precursors, which must make similar decisions regarding proliferation and migration, are similarly vulnerable to alcohol-mediated nucleolar stress and apoptosis [41]. The mechanism by which alcohol suppresses RBG is currently unknown. Ongoing work with O9-1 suggests they have an exceptional energy demand given their high proliferative rate. RBG typically occurs when cells are in an anabolic state [7, 8] and is stimulated by the major sensor of cellular anabolism, TORC, through its activation of RPS6 Kinase [71]. It is worth noting that alcohol can suppress TORC1 activity [72, 73]; moreover, exogenous L-leucine, an activator of TORC1, improves craniofacial development in alcohol-treated embryos [74].

The nucleolar stress described here is likely biologically relevant as it occurs at exposures as low as 20mM, which equates to a blood alcohol content of 0.08%, the legal limit for intoxication in the U.S. Moreover, this stress following a single 2hr exposure persists for 18-24hr. It is not surprising that the alcohol-mediated nucleolar stress does not fully recapitulate ribosomopathies of genetic origin, given that the alcohol exposure is transient and must target the narrow developmental window when premigratory CNC are vulnerable. Although genetic-based ribosomopathies are often characterized by anemia, those with PAE do not exhibit a persistent, postnatal anemia. However, fetal anemia has been reported in both rodent models of PAE and clinical assessments of cord blood from alcohol-exposed pregnancies [75, 76]. Although this fetal anemia is refractory to iron supplementation, the physical appearance of these erythrocytes is not wholly consistent with an iron-dependent anemia, and we have speculated that it might, in part, originate from a similar alcohol-induced nucleolar stress that limits the expansion of red blood cell pools within the fetal liver [77]. This hypothesis merits additional investigation.

In summary, we show that pharmacologically relevant alcohol exposure causes a nucleolar stress in CNCs that persists for at least 24hr after this exposure, and it is followed by their p53-MDM2 dependent apoptosis and craniofacial deficits that typify PAE. This apoptosis is presumably distinct from alcohol’s well characterized suppression of sonic hedgehog signaling, which targets gastrulation and determination of the head process field including the induction of CNC pools, whereas the events encompassed here occur slightly later in development during CNC expansion and migration; CNCs do not express sonic hedgehog, although those signals drive their further expansion within the facial anlage [78]. We note that the CNC apoptosis induced by gastrulation-stage alcohol exposure was recently shown to also be p53-dependent [34], raising the possibility that similar mechanisms may be relevant for that earlier stage; it is worth noting that gastrulation and head process formation are both typified by migration and proliferative expansion of the nascent germ layers. Our findings suggest that some facial dysmorphic features that characterize FASD may represent a ribosomopathy.

## Supporting information

S1 Table Reagents for studies. (PDF)

S2 Table Ribosome biogenesis-related genes dysregulated by alcohol. (PDF)

S1 Fig P53 western blot images. (PDF)
