# Supplementary material for: Alcohol exposure suppresses ribosome biogenesis and causes nucleolar stress in cranial neural crest cells
Source: PLoS One. 2024 Jun 28;19(6):e0304557. doi: 10.1371/journal.pone.0304557 (PMC11213321; doi:10.1371/journal.pone.0304557)
Supplement: S1 Table — (PDF) [file pone.0304557.s001.pdf]

S1 Table. Reagents for Studies

Morpholinos

Name	Conc.	Source	Sequence
Control Oligo	100 µM	Genetools[1]	5' – CCTCTTACCTCAGTTACAATTTAT - 3'
Tp53	5 µM	Genetools	5' – GCGCCATTGCTTTGCAAGAATTG - 3'
RPS3A	30 µM	Genetools	5' – TTTGCCGACTGCCATGTGAACAC - 3'
RPL5A	600 µM	Genetools	5' – ACCCATTTTGTGATCGTTTGTTCTC - 3'
RPL11	0.625 µM	Genetools	5' – CTTCTTCTCGCTCTGGTCCGCCATG - 3'
MO3-*nolc1*	0.1 µM	Genetools	5' – TAGGAACCGTGCTGTCCTCCGCCAT - 3'
MO1-*mdm2*	20 µM	Genetools	5' – CTCTGTTGCCATTTTGGTAGTTATC - 3'

Plasmids

Name	Concentration	Source	Catalog #
pCMV MDM2	0.01mg/ml	Addgene[2]	16441
XE150DN P53-pCS2P+	0.1 mg/ml	Addgene	17033
pCS2 (empty vector)	0.1 mg/ml		

Primary Antibodies

Name	Conc.	Source	Catalog #	Use
Coilin	1:500	Abcam[3]	210785	chick IHC*
Fibrillarin	1:500	Abcam	5821	chick, mouse IHC
Gapdh	1:2000	Sigma	G8795	mouse western blot
Nucleophosmin	1:100	Abcam	52644	chick, mouse IHC
Nucleolin	1:250	Abcam	22758	mouse IHC
p53	1:100	Santa Cruz	SC-99	chick IHC
p53, #135	1:50	N. Krupenko, UNC-Chapel Hill		mouse IHC
p53	1:500	Abcam	ab26	mouse western blot
UBF	1:250	Abcam	61205	mouse IHC

* IHC, immunohistochemistry

Primers for qPCR

Target	Source	Sequence	
Mouse ITS-1 (138 bp)	IDT[4]	Forward:	5'- CCGGCTTGCCCGATTT-3'
	IDT	Reverse:	5'- GCCAGCAGGAACGAAACG-3'
Mouse β2-microglobulin	IDT	Forward:	5'- TTCACCCCCACTGAGACTGAT-3'
(117 bp)	IDT	Reverse:	5'- GTCTTGGGCTCGGCCATA-3'
Chick ITS-1 (122bp)	IDT	Forward:	5'- CCGATATTCGTGTGCTCGTA-3'
	IDT	Reverse:	5'- CTCGTTCTCTCTCGCCTTG -3'
Chick β2-microglobulin	IDT	Forward:	5'- ACCAAGAACGTCCTCAACTG-3'
(117 bp)	IDT	Reverse:	5'- GTTGAAGGACATGTCGGAGTA-3'

[1] Genetools, LLC, Philomath OR
[2] Addgene, Watertown MA
[3] Abcam, Waltham MA
[4] IDT, Coralville IA
