# Supplementary material for: Alcohol exposure suppresses ribosome biogenesis and causes nucleolar stress in cranial neural crest cells
Source: PLoS One. 2024 Jun 28;19(6):e0304557. doi: 10.1371/journal.pone.0304557 (PMC11213321; doi:10.1371/journal.pone.0304557)
Supplement: S2 Table — (PDF) [file pone.0304557.s002.pdf]

Supplemental Table S2 - Ribosome Biogenesis-Related Genes Dysregulated by Alcohol

Gene ID	Gene	FC Alc/Con	P-adj	Wikigene description
**rRNA Synthesis - KEGG 03020 (13 genes)**				
ENSGALG00000007919	EIF4E2	0.747	9.41E-03	eukaryotic translation initiation factor 4E family member 2
ENSGALG00000003205	EIF4EBP1	0.700	2.78E-04	eukaryotic translation initiation factor 4E binding protein 1
ENSGALG00000006898	EIF4ENIF1	1.334	7.79E-02	eukaryotic translation initiation factor 4E nuclear import factor 1
ENSGALG00000015765	POLR1A	1.250	8.26E-02	polymerase (RNA) I polypeptide A, 194kDa
ENSGALG00000010349	POLR1C	0.716	1.84E-03	polymerase (RNA) I polypeptide C, 30kDa
ENSGALG00000007470	POLR1D	0.685	9.74E-03	polymerase (RNA) I polypeptide D
ENSGALG00000010863	POLR1F	0.768	7.35E-02	polymerase (RNA) I polypeptide F (TWIST neighbor)
ENSGALG00000004947	POLR3A	1.362	4.13E-02	polymerase (RNA) III (DNA directed) polypeptide A, 155kDa
ENSGALG00000008749	POLR3F	0.800	9.84E-02	polymerase (RNA) III (DNA directed) polypeptide F, 39 kDa
ENSGALG00000011947	POLR3H	0.745	1.42E-02	polymerase (RNA) III (DNA directed) polypeptide H, 22.9kD
ENSGALG00000017681	POLR3K	0.464	1.15E-03	polymerase (RNA) III (DNA directed) polypeptide K, 12.3 kDa
ENSGALG00000007097	RPS6KA6	1.976	7.33E-05	ribosomal protein S6 kinase, 90kDa, polypeptide 6
ENSGALG00000022512	RPS6KB2	0.656	7.41E-02	ribosomal protein S6 kinase beta-2-like
**rRNA Processing & Assembly - KEGG 03008 (61 genes)**				
ENSGALG00000006197	CSNK2A1	0.791	2.39E-02	casein kinase 2, alpha 1 polypeptide
ENSGALG00000021251	DDX6	1.653	1.59E-05	DEAD (Asp-Glu-Ala-Asp) box polypeptide 6
ENSGALG00000012247	DDX17	1.366	1.21E-03	DEAD (Asp-Glu-Ala-Asp) box polypeptide 17
ENSGALG00000010235	DDX19B	0.768	7.21E-03	DEAD (Asp-Glu-Ala-Asp) box polypeptide 19B
ENSGALG00000000463	DDX25	0.733	5.51E-02	DEAD (Asp-Glu-Ala-Asp) box polypeptide 25
ENSGALG00000006974	DDX27	0.815	6.09E-02	DEAD (Asp-Glu-Ala-Asp) box polypeptide 27
ENSGALG00000027656	DDX41	0.761	6.49E-02	DEAD (Asp-Glu-Ala-Asp) box polypeptide 41
ENSGALG00000005489	DDX52	0.771	4.54E-02	DEAD (Asp-Glu-Ala-Asp) box polypeptide 52
ENSGALG00000007490	DDX54	0.751	1.87E-02	DEAD (Asp-Glu-Ala-Asp) box polypeptide 54
ENSGALG00000005054	DKC1	0.826	7.46E-02	dyskerin, H/ACA ribonucleoprotein complex subunit 4
ENSGALG00000008187	EIF3J	0.734	4.39E-03	eukaryotic translation initiation factor 3, subunit J
ENSGALG00000014568	EMG1	0.669	8.49E-04	EMG1 nucleolar protein homolog (S. cerevisiae)
ENSGALG00000010275	FCF1	0.776	2.77E-02	FCF1 small subunit (SSU) processome component homolog (S. cerevisiae)
ENSGALG00000000332	FTSJ3	0.708	4.34E-04	FtsJ homolog 3 (E. coli), putative rRNA methyltransferase 3
ENSGALG00000020164	GAR1	0.750	2.58E-02	GAR1 ribonucleoprotein homolog (yeast) (H/ACA snoRNP)
ENSGALG00000026786	GNL1	0.588	4.29E-03	G protein nucleolar 1 (putative)
ENSGALG00000001613	GNL3	0.789	2.05E-02	guanine nucleotide binding protein-like 3 (nucleostemin)
ENSGALG00000006703	GTPBP4	0.793	2.94E-02	nucleolar GTP binding protein 4
ENSGALG00000029152	IMP4	0.650	5.77E-05	U3 small nucleolar ribonucleoprotein protein, IMP4-like
ENSGALG00000004001	LARP1	1.697	9.35E-04	La ribonucleoprotein domain family, member 1
ENSGALG00000028166	LOC100857591	0.586	9.92E-05	probable ATP-dependent RNA helicase DDX23-like
ENSGALG00000007058	LSG1	0.783	2.60E-02	large 60S subunit nuclear export GTPase 1 homolog (S. cerevisiae)
ENSGALG00000011650	MKI67IP	0.814	8.57E-02	MKI67 (FHA domain) interacting nucleolar phosphoprotein (NIFK)
ENSGALG00000000693	NOB1	0.578	4.70E-08	NIN1/RPN12 binding protein 1 homolog (S. cerevisiae)
ENSGALG00000012695	NOL7	0.681	2.50E-03	nucleolar protein 7, 27kDa
ENSGALG00000028958	NOL9 (LAS1L)	0.734	1.82E-03	nucleolar protein 9
ENSGALG00000012410	NOL12	0.728	8.55E-03	nucleolar protein 12
ENSGALG00000028109	NOP9	0.612	4.74E-03	NOP9 ribonucleoprotein homolog (yeast)
ENSGALG00000003355	NOP16	0.590	5.54E-07	NOP16 nucleolar protein homolog (yeast)
ENSGALG00000014498	NOP56	0.617	2.98E-07	NOP56 ribonucleoprotein homolog (yeast)
ENSGALG00000008454	NOP58	0.742	6.29E-03	NOP58 ribonucleoprotein homolog (yeast)
ENSGALG00000002197	NPM1	0.762	4.16E-03	nucleophosmin (nucleolar phosphoprotein B23, numatrin)
ENSGALG00000007704	NPM3	0.581	2.15E-09	nucleophosmin/nucleoplasmin 3
ENSGALG00000010360	NSUN4	0.717	1.08E-02	NOP2/SUN RNA methyltransferase family, member 4
ENSGALG00000000891	NSUN5	0.616	1.39E-04	NOP2/SUN RNA methyltransferase family, member 5
ENSGALG00000007778	PES1	0.760	6.29E-03	pescadillo homolog 1, containing BRCT domain (zebrafish)
ENSGALG00000009093	PNO1	0.668	1.73E-04	partner of NOB1 homolog (S. cerevisiae)
ENSGALG00000007124	POP5	0.497	5.75E-08	processing of precursor 5, ribonuclease P/MRP subunit (S. cerevisiae)
ENSGALG00000007835	PUS10	1.375	3.05E-02	pseudouridylate synthase 10
ENSGALG00000002569	RAN	0.746	2.32E-03	RAN, member RAS oncogene family
ENSGALG00000016808	RANBP2	1.231	5.82E-02	RAN binding protein 2
ENSGALG00000002184	RANBP17	1.389	4.43E-02	ran-binding protein 17-like
ENSGALG00000007024	REXO2	0.703	1.71E-03	REX2, RNA exonuclease 2 homolog (S. cerevisiae)
ENSGALG00000015043	RPF2	0.813	7.99E-02	ribosome production factor 2 homolog (S. cerevisiae)
ENSGALG00000024326	RPUSD4	0.715	8.85E-03	RNA pseudouridylate synthase domain containing 4 (mitochondrial)
ENSGALG00000000576	RRNAD1	0.618	1.77E-02	ribosomal RNA adenine dimethylase domain containing 1
ENSGALG00000011866	RRP7A	0.721	8.48E-03	ribosomal RNA processing 7 homolog A (S. cerevisiae)
ENSGALG00000028679	RRP8	0.689	1.52E-02	ribosomal RNA processing 8, methyltransferase, homolog (yeast)
ENSGALG00000001422	RRP9	0.605	1.18E-07	ribosomal RNA processing 9, small subunit (SSU) processome component, homolog (yeast)
ENSGALG00000007603	RRP12	0.807	5.16E-02	ribosomal RNA processing 12 homolog (S. cerevisiae)
ENSGALG00000005558	TBL3	0.803	4.54E-02	transducin (beta)-like 3
ENSGALG00000005535	TCOF1	0.727	1.07E-03	Treacher Collins-Franceschetti syndrome 1
ENSGALG00000003180	TSR1	0.784	2.56E-02	pre-rRNA-processing protein TSR1 homolog
ENSGALG00000011553	UTP3	0.691	4.82E-04	UTP3, small subunit (SSU) processome component, homolog (S. cerevisiae)
ENSGALG00000000638	UTP4 (CIRH1A)	0.624	1.26E-06	UTP4 small subunit processome component; cirrhosis, autosomal recessive 1A (cirhin)
ENSGALG00000003260	UTP6	0.783	4.62E-02	UTP6, U3 small subunit (SSU) processome component, homolog (yeast)
ENSGALG00000001502	UTP11L	0.780	4.05E-02	UTP11-like, U3 small nucleolar ribonucleoprotein, (yeast)
ENSGALG00000024039	UTP14A	0.642	3.12E-05	UTP14, U3 small nucleolar ribonucleoprotein, homolog A (yeast)
ENSGALG00000016120	UTP23	0.672	4.17E-02	UTP23, small subunit (SSU) processome component, homolog (yeast)

ENSGALG00000008989	WDR43	0.832	9.84E-02	WD repeat domain 43
ENSGALG00000004377	XPO1	1.614	2.13E-07	exportin 1 (CRM1 homolog, yeast)
ENSGALG00000002706	XRN1	1.314	2.85E-02	5'-3' exoribonuclease 1

Ribosomal Proteins - KEGG 04150 (106 genes, 66 nuclear + 40 mitochondrial)

ENSGALG00000012172	RPL3	0.779	9.97E-03	ribosomal protein L3
ENSGALG00000007711	RPL4	0.691	4.91E-05	ribosomal protein L4
ENSGALG00000005922	RPL5	0.759	3.75E-03	ribosomal protein L5
ENSGALG00000004818	RPL6	0.703	1.18E-04	ribosomal protein L6
ENSGALG00000015637	RPL7	0.669	1.94E-04	ribosomal protein L7
ENSGALG00000003197	RPL7A	0.699	9.45E-05	ribosomal protein L7a
ENSGALG00000016232	RPL8	0.704	1.36E-04	ribosomal protein L8
ENSGALG00000000150	RPL9	0.683	3.23E-05	ribosomal protein L9
ENSGALG00000027227	RPL10L	0.495	3.54E-15	ribosomal protein L10-like
ENSGALG00000002644	RPL10A	0.648	3.00E-05	ribosomal protein L10a
ENSGALG00000003971	RPL11	0.694	7.52E-02	ribosomal protein L11
ENSGALG00000008806	RPL12	0.607	2.02E-08	ribosomal protein L12
ENSGALG00000006179	RPL13	0.587	1.71E-09	ribosomal protein L13
ENSGALG00000011523	RPL14	0.553	4.43E-06	ribosomal protein L14
ENSGALG00000011290	RPL15	0.619	6.59E-08	ribosomal protein L15
ENSGALG00000022174	RPL17L	0.551	2.04E-08	ribosomal protein 17-like
ENSGALG00000009829	RPL18A	0.637	6.58E-07	ribosomal protein L18a
ENSGALG00000001658	RPL19	0.547	4.71E-12	ribosomal protein L19
ENSGALG00000027035	RPL21	0.639	1.99E-02	ribosomal protein L21
ENSGALG00000000719	RPL22	0.544	8.37E-12	ribosomal protein L22
ENSGALG00000009312	RPL22L1	0.687	6.24E-03	ribosomal protein L22-like 1
ENSGALG00000001634	RPL23	0.558	5.32E-07	ribosomal protein L23
ENSGALG00000003966	RPL23A	0.567	1.11E-10	ribosomal protein L23a
ENSGALG00000015339	RPL24	0.614	4.13E-03	ribosomal protein L24
ENSGALG00000002868	RPL26	0.614	4.26E-08	ribosomal protein L26-like
ENSGALG00000002837	RPL27	0.588	1.71E-09	ribosomal protein L27
ENSGALG00000005948	RPL27A	0.584	1.26E-09	ribosomal protein L27a
ENSGALG00000026978	RPL29	0.422	9.66E-05	ribosomal protein L29
ENSGALG00000008212	RPL30	0.560	3.09E-10	ribosomal protein L30
ENSGALG00000016775	RPL31	0.604	1.41E-08	ribosomal protein L31
ENSGALG00000027142	RPL32	0.599	6.59E-09	ribosomal protein L32
ENSGALG00000028992	RPL34L	0.702	1.68E-02	ribosomal protein L34-like
ENSGALG00000001039	RPL35	0.512	1.00E-02	ribosomal protein L35
ENSGALG00000007611	RPL35A	0.582	2.08E-05	ribosomal protein L35a
ENSGALG00000000474	RPL36	0.358	3.22E-08	ribosomal protein L36
ENSGALG00000004952	RPL36A	0.582	3.47E-05	ribosomal protein L36a
ENSGALG00000014833	RPL37	0.611	3.21E-08	ribosomal protein L37
ENSGALG00000011472	RPL37A	0.543	4.30E-12	ribosomal protein L37a
ENSGALG00000001465	RPL38	0.520	2.36E-13	ribosomal protein L38
ENSGALG00000008620	RPL39	0.639	5.18E-02	ribosomal protein L39
ENSGALG00000023294	RPLP0	0.614	3.49E-08	ribosomal protein, large, P0
ENSGALG00000016172	RPLP1	0.608	5.78E-03	ribosomal protein, large, P1
ENSGALG00000014309	RPLP2	0.586	1.32E-02	ribosomal protein, large, P2
ENSGALG00000005490	RPS2	0.644	6.72E-07	ribosomal protein S2
ENSGALG00000017330	RPS3	0.612	8.67E-08	ribosomal protein S3
ENSGALG00000010077	RPS3A	0.708	1.52E-04	ribosomal protein S3A
ENSGALG00000004831	RPS4	0.634	3.23E-07	ribosomal protein S4, X-linked
ENSGALG00000015082	RPS6	0.777	9.06E-03	ribosomal protein S6
ENSGALG00000010124	RPS8	0.599	5.10E-09	ribosomal protein S8
ENSGALG00000002813	RPS10	0.591	2.23E-09	ribosomal protein S10
ENSGALG00000017299	RPS11	0.587	1.87E-09	ribosomal protein S11
ENSGALG00000013990	RPS12	0.593	2.58E-09	ribosomal protein S12
ENSGALG00000006096	RPS13	0.578	6.39E-03	ribosomal protein S13
ENSGALG00000004588	RPS14	0.666	7.70E-06	ribosomal protein S14
ENSGALG00000015195	RPS15	0.509	3.20E-03	ribosomal protein S15
ENSGALG00000006771	RPS15A	0.588	1.91E-02	ribosomal protein S15a
ENSGALG00000026490	RPS16	0.634	1.23E-04	ribosomal protein S16
ENSGALG00000002157	RPS17	0.372	1.59E-12	ribosomal protein S17
ENSGALG00000014432	RPS20	0.616	1.71E-03	ribosomal protein S20
ENSGALG00000015617	RPS23	0.801	2.91E-02	ribosomal protein S23
ENSGALG00000004871	RPS24	0.617	4.10E-04	ribosomal protein S24
ENSGALG00000007699	RPS25	0.595	4.89E-08	ribosomal protein S25
ENSGALG00000027807	RPS26	0.561	4.50E-04	ribosomal protein S26
ENSGALG00000008125	RPS27A	0.621	5.40E-04	ribosomal protein S27a
ENSGALG00000024398	RPS28	0.511	3.49E-11	ribosomal protein S28
ENSGALG00000012229	RPS29	0.568	8.67E-08	ribosomal protein S29
ENSGALG00000011951	RPSA	0.708	1.65E-04	ribosomal protein SA
ENSGALG00000008597	MRPL2	0.527	1.60E-09	mitochondrial ribosomal protein L2
ENSGALG00000025814	MRPL9	0.294	1.55E-10	mitochondrial ribosomal protein L9
ENSGALG00000005182	MRPL16	0.495	2.11E-07	mitochondrial ribosomal protein L16
ENSGALG00000002605	MRPL17	0.682	7.56E-04	mitochondrial ribosomal protein L17
ENSGALG00000011625	MRPL18	0.690	7.71E-04	mitochondrial ribosomal protein L18
ENSGALG00000016731	MRPL19	0.738	1.25E-02	mitochondrial ribosomal protein L19
ENSGALG00000001542	MRPL20	0.699	7.63E-04	mitochondrial ribosomal protein L20

ENSGALG00000006561	MRPL23	0.624	1.96E-03	mitochondrial ribosomal protein L23
ENSGALG00000013242	MRPL24	0.509	1.85E-10	mitochondrial ribosomal protein L24
ENSGALG00000007473	MRPL28	0.659	8.16E-02	mitochondrial ribosomal protein L28
ENSGALG00000016760	MRPL30	0.694	5.17E-03	mitochondrial ribosomal protein L30
ENSGALG00000022950	MRPL35	0.620	6.09E-02	mitochondrial ribosomal protein L35
ENSGALG00000010761	MRPL37	0.773	4.80E-02	mitochondrial ribosomal protein L37
ENSGALG00000002194	MRPL38	0.776	3.05E-02	mitochondrial ribosomal protein L38
ENSGALG00000015742	MRPL39	0.740	1.35E-02	mitochondrial ribosomal protein L39
ENSGALG00000001587	MRPL40	0.757	2.96E-02	mitochondrial ribosomal protein L40
ENSGALG00000008746	MRPL41	0.462	2.37E-05	mitochondrial ribosomal protein L41
ENSGALG00000003067	MRPL44	0.615	1.87E-04	mitochondrial ribosomal protein L44
ENSGALG00000006782	MRPL46	0.613	1.05E-05	mitochondrial ribosomal protein L46
ENSGALG00000013759	MRPL47	0.668	3.00E-02	mitochondrial ribosomal protein L47
ENSGALG00000017319	MRPL48	0.729	5.10E-03	mitochondrial ribosomal protein L48
ENSGALG00000015543	MRPL50	0.753	6.86E-02	mitochondrial ribosomal protein L50
ENSGALG00000014435	MRPL51	0.678	6.11E-02	mitochondrial ribosomal protein L51
ENSGALG00000027611	MRPL54	0.568	5.47E-02	mitochondrial ribosomal protein L54
ENSGALG00000027824	MRPL55	0.404	6.00E-09	mitochondrial ribosomal protein L55
ENSGALG00000026303	MRPS2	0.662	2.62E-04	mitochondrial ribosomal protein S2
ENSGALG00000008550	MRPS5	0.740	4.51E-03	mitochondrial ribosomal protein S5
ENSGALG00000007999	MRPS7	0.798	7.69E-02	mitochondrial ribosomal protein S7
ENSGALG00000009890	MRPS10	0.795	8.49E-02	mitochondrial ribosomal protein S10
ENSGALG00000006777	MRPS11	0.690	9.80E-04	mitochondrial ribosomal protein S11
ENSGALG00000022193	MRPS12	0.601	4.51E-02	mitochondrial ribosomal protein S12
ENSGALG00000026803	MRPS14	0.646	2.13E-04	mitochondrial ribosomal protein S14
ENSGALG00000027648	MRPS15	0.599	1.38E-06	mitochondrial ribosomal protein S15
ENSGALG00000023191	MRPS18C	0.575	7.59E-03	mitochondrial ribosomal protein S18C
ENSGALG00000000456	MRPS21	0.457	3.75E-14	mitochondrial ribosomal protein S21
ENSGALG00000005367	MRPS22	0.716	1.76E-03	mitochondrial ribosomal protein S22
ENSGALG00000005559	MRPS23	0.654	8.41E-05	mitochondrial ribosomal protein S23
ENSGALG00000008506	MRPS25	0.698	5.81E-02	mitochondrial ribosomal protein S25
ENSGALG00000014118	MRPS26	0.763	1.20E-02	mitochondrial ribosomal protein S26
ENSGALG00000001792	MRPS34	0.716	1.66E-03	mitochondrial ribosomal protein S34

tRNA Synthesis - KEGG 00970 (27 genes)

ENSGALG00000002880	AARSD1	0.661	4.26E-04	alanyl-tRNA synthetase domain containing 1
ENSGALG00000010553	AIMP1	0.797	5.17E-02	aminoacyl tRNA synthetase complex-interacting multifunctional protein 1
ENSGALG00000003400	AIMP2	0.788	3.68E-02	aminoacyl tRNA synthetase complex-interacting multifunctional protein 2
ENSGALG00000001800	DUS3L	0.724	7.42E-03	dihydrouridine synthase 3-like (S. cerevisiae)
ENSGALG00000006122	EARS2	0.672	3.93E-03	glutamyl-tRNA synthetase 2, mitochondrial (putative)
ENSGALG00000025990	FARSA	0.541	2.23E-09	phenylalanyl-tRNA synthetase, alpha subunit
ENSGALG00000007195	GATC	0.753	6.77E-02	glutamyl-tRNA(Gln) amidotransferase, subunit C homolog (bacterial)
ENSGALG00000000794	HARS	0.774	1.71E-02	histidyl-tRNA synthetase
ENSGALG00000009566	IARS2	0.822	8.29E-02	isoleucyl-tRNA synthetase 2, mitochondrial
ENSGALG00000000907	KARS	0.732	1.15E-03	lysyl-tRNA synthetase
ENSGALG00000028714	MARS	0.638	1.03E-03	methionyl-tRNA synthetase
ENSGALG00000015923	MTO1	0.759	1.63E-02	mitochondrial translation optimization 1 homolog (S. cerevisiae)
ENSGALG00000017262	NARS2	0.788	9.64E-02	asparaginyl-tRNA synthetase 2, mitochondrial (putative)
ENSGALG00000010789	PARS2	0.672	5.21E-03	prolyl-tRNA synthetase 2, mitochondrial (putative)
ENSGALG00000007124	POP5	0.497	5.75E-08	processing of precursor 5, ribonuclease P/MRP subunit (S. cerevisiae)
ENSGALG00000009610	PSTK	0.649	6.11E-03	phosphoseryl-tRNA kinase
ENSGALG00000005150	PTRH2	0.640	1.19E-03	peptidyl-tRNA hydrolase 2, mitochondrial
ENSGALG00000016610	PTRHD1	0.712	3.99E-02	peptidyl-tRNA hydrolase domain containing 1
ENSGALG00000006853	QARS	0.641	1.86E-04	glutaminyl-tRNA synthetase
ENSGALG00000001850	RARS	0.698	1.52E-04	arginyl-tRNA synthetase
ENSGALG00000026809	SARS	0.677	1.04E-03	seryl-tRNA synthetase
ENSGALG00000028719	SARS2	0.621	8.47E-04	seryl-tRNA synthetase, mitochondrial-like
ENSGALG00000014386	SEPSECS	1.261	7.74E-02	Sep (O-phosphoserine) tRNA:Sec (selenocysteine) tRNA synthase
ENSGALG00000026218	TARS2	0.655	4.10E-05	threonyl-tRNA synthetase, mitochondrial-like
ENSGALG00000014831	TRMT11	1.462	4.79E-02	tRNA guanosine-2'-O-methyltransferase 11 homolog (S. cerevisiae)
ENSGALG00000009835	XPOT	1.240	8.46E-02	exportin, tRNA (nuclear export receptor for tRNAs)
ENSGALG00000003563	YARS	0.709	1.28E-03	tyrosyl-tRNA synthetase

P53 Signaling

ENSGALG00000017159	ATM	1.383	1.11E-02	ataxia telangiectasia mutated
ENSGALG00000003254	BAG4	0.787	5.93E-02	BCL2-associated athanogene 4
ENSGALG00000006211	BCL2L1	0.780	5.10E-02	BCL2-like 1
ENSGALG00000026951	BCL7B	0.760	5.29E-02	B-cell CLL/lymphoma 7B
ENSGALG00000003788	BNIP2	1.381	8.89E-02	BCL2/adenovirus E1B 19kDa interacting protein 2
ENSGALG00000004161	CCNB2	0.752	3.70E-03	cyclin B2
ENSGALG00000025810	CCNB3	0.624	8.85E-07	cyclin B3
ENSGALG00000017283	CCND2	1.384	2.51E-02	cyclin D2
ENSGALG00000004837	CCNDBP1	0.785	4.98E-02	cyclin D-type binding-protein 1
ENSGALG00000009476	CDK6	0.699	8.37E-02	cyclin-dependent kinase 6
ENSGALG00000001613	GNL3	0.789	2.05E-02	guanine nucleotide binding protein-like 3 (nucleolar)
ENSGALG00000009942	MDM2	1.468	3.27E-03	Mdm2 p53 binding protein homolog (mouse)
ENSGALG00000000636	MDM4	1.963	8.17E-08	Mdm4 p53 binding protein homolog (mouse)
ENSGALG00000006426	PAK2	1.643	3.78E-04	p21 protein (Cdc42/Rac)-activated kinase 2
ENSGALG00000008058	PAK3	1.734	3.56E-04	p21 protein (Cdc42/Rac)-activated kinase 3

ENSGALG00000014303	PAK4	0.669	1.76E-02	p21 protein (Cdc42/Rac)-activated kinase 4
ENSGALG00000017706	RASA1	1.317	1.46E-02	RAS p21 protein activator (GTPase activating protein) 1
ENSGALG00000001876	RASA4	0.595	1.22E-04	RAS p21 protein activator 4
ENSGALG00000004323	RASAL2	1.322	5.98E-02	RAS protein activator like 2
ENSGALG00000012132	RPS19BP1	0.745	3.19E-02	ribosomal protein S19 binding protein 1
ENSGALG00000016049	RRM2B	1.542	6.19E-03	ribonucleotide reductase M2 B (TP53 inducible)
ENSGALG00000011619	SIVA1	0.692	4.09E-03	SIVA1, apoptosis-inducing factor
ENSGALG00000014069	TP53I11	1.437	4.82E-02	tumor protein p53 inducible protein 11
ENSGALG00000015987	TP53INP1	1.275	3.97E-02	tumor protein p53 inducible nuclear protein 1
ENSGALG00000004469	TP53RK	0.662	7.13E-03	TP53 regulating kinase
