# Supplementary figures and images for: Alcohol exposure suppresses ribosome biogenesis and causes nucleolar stress in cranial neural crest cells
Source: PLoS One. 2024 Jun 28;19(6):e0304557. doi: 10.1371/journal.pone.0304557 (PMC11213321; doi:10.1371/journal.pone.0304557)

# p35 western blots

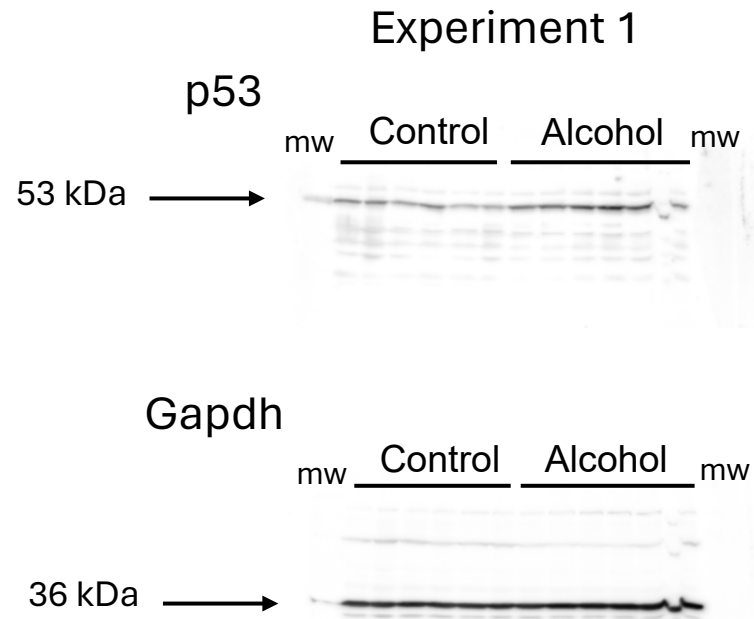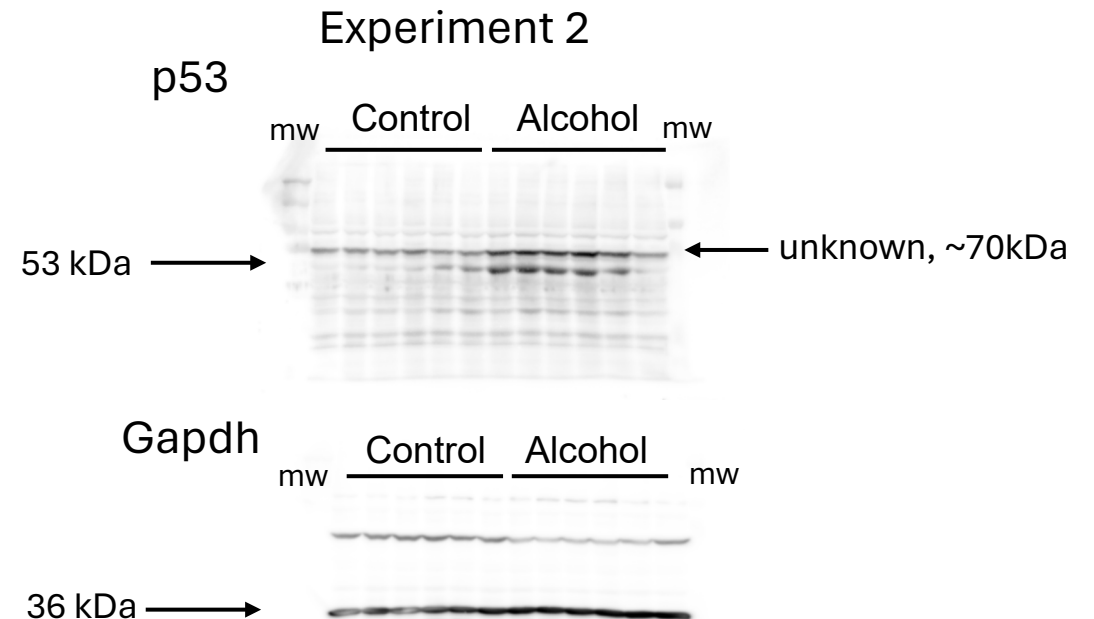

Supplement: S1 Fig — (PDF) [file pone.0304557.s003.pdf]
